# Provenance, paleoweathering, depositional setting and paleoclimatic constraints of cretaceous and neogene deposits of The Mamfe Basin, southwest Cameroon

**DOI:** 10.1016/j.heliyon.2022.e10304

**Published:** 2022-08-27

**Authors:** Jeanne Armelle Bilobé, John Takem Eyong, Elias Samankassou

**Affiliations:** a Department of Earth Sciences, University of Geneva, 13 Rue des Maraîchers, 1205 Geneva, Switzerland; b Department of Earth Sciences, University of Yaounde I, P.O. Box 812, Yaounde, Cameroon

**Keywords:** Provenance, Paleoweathering, Depositional environment, Paleoclimate, Mamfe basin

## Abstract

The Cretaceous and Neogene deposits from the Mamfe Basin consisting of sandstone, shale and claystone were studied using petrography, and major, traces and REEs analyses to address sediment source, environment setting, prevailing paleoclimate as well as tectonic regime of the basin. The angular to subangular shape of detrital grains reflects the mineralogical and textural immaturity of sediments and the proximity of the sediment supply source. Sedimentary rocks are composed of a significant number of lithic debris, organic matter, ostracods as well as subrounded heavy minerals referring to notable igneous and metamorphic rocks bordering the Mamfe Basin. The plots of major element ratios including iron oxide/potasium oxide (Fe_2_O/K_2_O) against silicium oxide/aluminium oxide (SiO_2_/Al_2_O_3_) combined with that of sodium oxide/potassium oxide (Na_2_O/K_2_O) compared to silicium oxide/aluminium oxide (SiO_2_/Al_2_O_3_) are characteristic of greywacke and shale with few arkoses. The pronounced Eu negative anomaly of chondrite normalized REEs along with the plot of La/Th vs Hf and Co/Th vs La/Sc suggest that sediments are in general from felsic and intermediate source rock provenance, only subordinated contribution of mafic source. The negative anomaly of Yb suggests igneous fractionation under highly reducing conditions. The chemical index of alteration values of 47–70 combined with chemical index of weathering values of 0.6–84 suggest low to moderate weathering process of the sediment in the basin. This result is further confirmed by an index of chemical variability values of 0.6–100 and Zr/Sc ratio of 0.06–2.96. The REEs distribution displays a substantial content in LREE, low content in HREE and noticible proportion of (La/Yb)_N_ ratio (mean >9), poor (Gd/Yb)_N_ ratio in the Cross River Formation (mean <2) and slightly moderate (Gd/Yb)_N_ ratio in the other formations (mean >2). This result implies that sediments from the Ngeme, Nfaitok and Baso formations derived from post-Archean rocks. Geochemical paleoenvironmental proxies including Sr/Cu, Sr/Ba, Ga/Rb vs Sr/Cu and SiO_2_ vs K_2_O + NaO_2_+Al_2_O_3_ are in favor of arid to semi-arid conditions during the deposition. Trace Elemental ratios such as Sr/Cu, Sr/Ba, V/Ni, U/Th, Ni/Co, V/Sc, and V/Cr values indicate a predominance of oxic conditions during deposition. In contrast, some authigenic pyrite, hematite, siderite and vivianite which are iron-rich minerals suggests episodic reducing conditions in the basin. The study provides a valuable information in evaluating sediments source, depositional environment, tectonic regime as well as the paleoclimatic conditions prevailing in the basin during the depositional period. The geochemistry of rocks of the Ngeme and Baso formations suggest passive continental margin setting and Ngeme, Nfaitok and Cross River formations suggest oceanic island Arc tectonic setting.

## 1 Introduction

**Figure 1:**
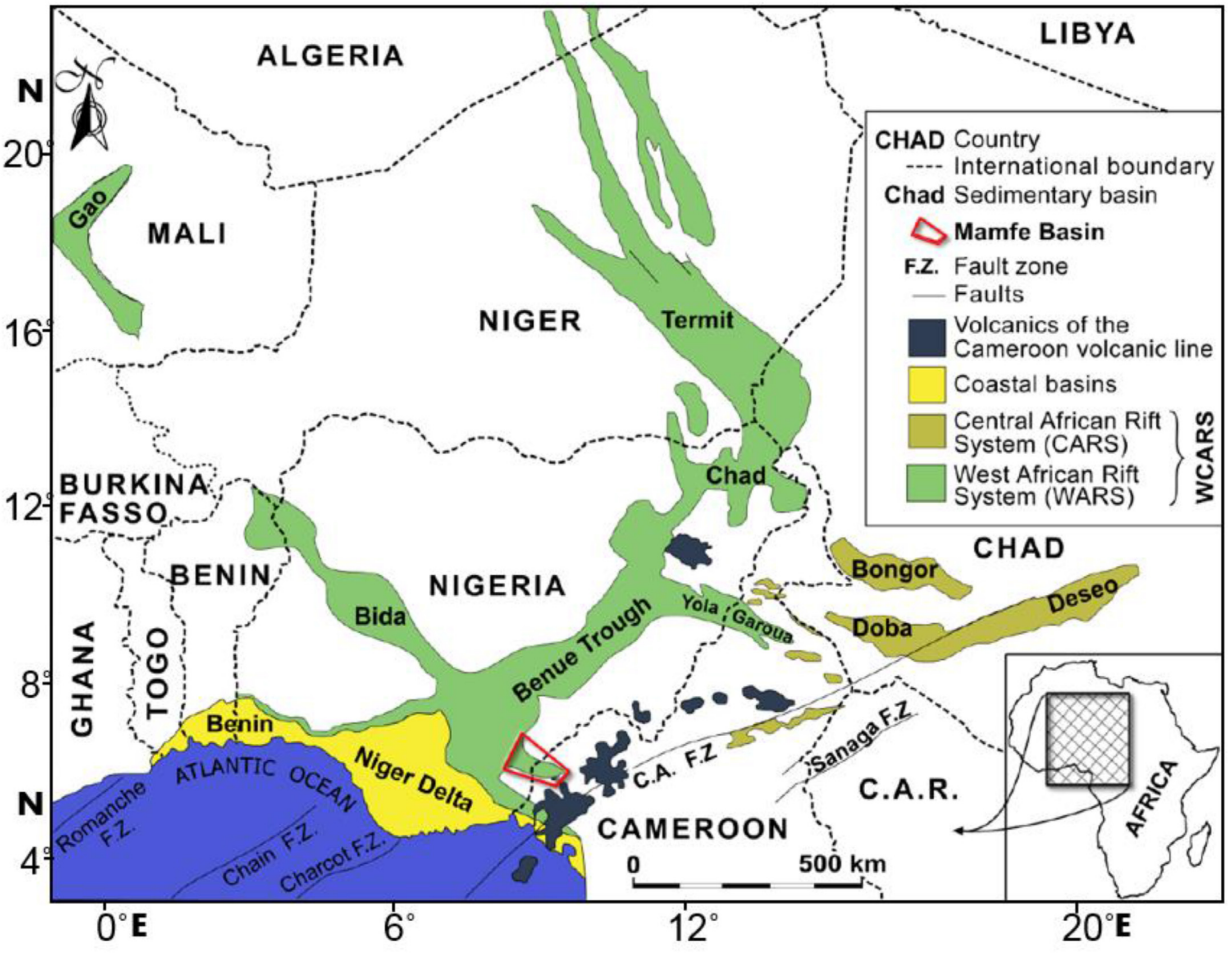
Location map showing the West and Central African rift systems (WCARS) displaying the relationship between the Mamfe Basin, the focus of the present study, and the adjacent basins (modified from Genick, 1993).

The Mamfe sedimentary basin (MSB) represents an inland basin located in southwest Cameroon, recording with a Cretaceous and *Neogene infill*. The basin represents one of the several branches of the Benue Trough of Nigeria, covering Chad, Niger, Benin and Cameroon (Figure 1). The basin is known as a rift formed in response to the Gondwana dislocation and further detachment of the later South American and African plates in the Early Cretaceous time (Ndougsa Mbarga, 2004; Abolo, 2008; Nguimbous-kouoh et al., 2012; Njoh et al., 2015). The elliptical basin covers an area as large as 2400 km^2^, with a length of 130 km and width of 60 km and forms a close expansion of the south Benue Trough of Nigeria (Eyong, 2003; Abolo, 2008; Njoh and Njie, 2016; Ajonina, 2016).

Cameroonian coastal basins including Rio del Rey Basin and Douala/Kribi/Campo Basin have attracted earlier workers in exploration geology due to their economic importance in contrast to intracratonic basins as in the case of Mamfe Basin. Inferred Various depositional environments were inferred for the MSB, including fluvial, deltaic, lacustrine, marine and estuarine (Petters, 1991, 1995; Tanyileke et al., 1996; Tijani et al., 1996; Ajonina and Bassey, 1997; Ajonina et al., 1998, 2001; Eyong, 2003; Eseme et al., 2006). Previous geochemical studies in Mamfe Basin are based on the brine emanations origins (Eseme et al., 2002), grey black rutile of Nsanaragati (Kanouo et al., 2012) and depositional setting and oxidation state (Bokanda et al., 2018). Furthermore, prior geochemical studies have been focused to the central and southwest part of the basin, thus questions regarding provenance, paleoweathering, depositional environment and paleoclimate in the basin remain partially explored.

This paper uses sedimentologic, inorganic geochemistry of major, traces and REEs, as well as petrography and statistical tools to infer provenance, paleoweathering, mineral composition, depositional environment and paleoclimate conditions during the depositional history to characterize the sedimentation conditions in the MSB. Our data sets provide comprehensive and detailed insights into diverse mineral compositions and their spatial distribution in the basin and supply new data to refine knowledge of the Mamfe Basin evolution.

## 2 Geological setting

The MSB is the southeastern branch of the Lower Benue Trough, which trends from WNW-ESE (Figure 1). The basin is limited to the south by the Oban Massif, a Precambrian Basement that splits the Mamfe Basin from the Rio del Rey Basin and the north by the Precambrian rocks of the Obudu Massif. The west of the MSB is open and extends as a part of southeastern Benue Trough of Nigeria, a large SW-NE oriented intracontinental rift belonging to the West and Central African Rift System (WCARS) (Petters and Okereke, 1987). The Trough shrinks and ends at the Cameroon Volcanic Line (CVL) in the northeastern part (Njoh et al., 2015). Moreover, the Mamfe Basin in general is situated in the north-east of the Gulf of Guinea, at 200 km from the Atlantic coast and around 150 km northeast of the Niger Delta Basin (Ajonina, 2016). The Benue Trough expands in southeastern to the Gulf of Guinea, in the north into Niger and Libya and eastwards into Chad and Sudan and its southeasterly trending branch into southwestern Cameroon (Ajonina, 2016).

The Mamfe Basin infill started after the Gondwana breakup during the Early Cretaceous, in the Albian according to Dumort (1968). Le Fur (1965) described five lithologic series designated cg1 to cg5 in the basin. Subsequent studies estimated sediment infill of the MSB to exceed 4500 m thickness (Petters and Okereke, 1987; Hell et al., 2000; Heine, 2007) lying unconformably on the Pan-African granito-gneissic Basement. Precambrian Basement rocks of the MSB comprise granites, migmatites, gneisses and schists, which are altered to form a bedrock for Cretaceous and Neogene sediments before the end of volcanic activities. Those rocks are from reorganisation of the native craton, related to the Pan-African movement (550 ± 100 Ma). The most important strike of the rocks is oriented E-W with sporadic swings to the N–S (Wilson, 1928; Dumort, 1968; Regnoult, 1986). Early Cretaceous sediments deposited in the east are deformed into an atypical anticline layered by a flat unit to the west deposited during the mid-Cretaceous. While the sediments were later crosscut by syenites, diorites covered by rhyolites, trachytes, basalts and volcaniclastics of Cenozoic age (Bilobe et al., 2021).

The north-eastern part of the basin margins displays normal faults characterized by breccias and conglomerates. The sedimentary rocks in the basin consist of conglomerates, sandstone, siltstones, limestone, mudstones, clay shales, carbonate shales, coal and volcaniclastics. Additionally, there are also crystalline rocks such as basalts, diorites, granite and porphyroid granite (Bilobe et al., 2021).

Three tectono-stratigraphic depositional sequences were defined by Eyong et al. (2018) corresponding to the pre-rift, syn-rift to post-rift phases. These three sequences were subdivided into four lithostratigraphic formations namely Ngeme, Nfaitok, Baso and Cross River formations, considered to be set up by various deposition settings. Bilobe et al. (2021) identified a new Cenozoic horizon in the basin characterized by Pleistocene and Miocene diversified palynomorphs whose formation affiliation remain questionable.

## 3 Analytical methods

**Figure 2:**
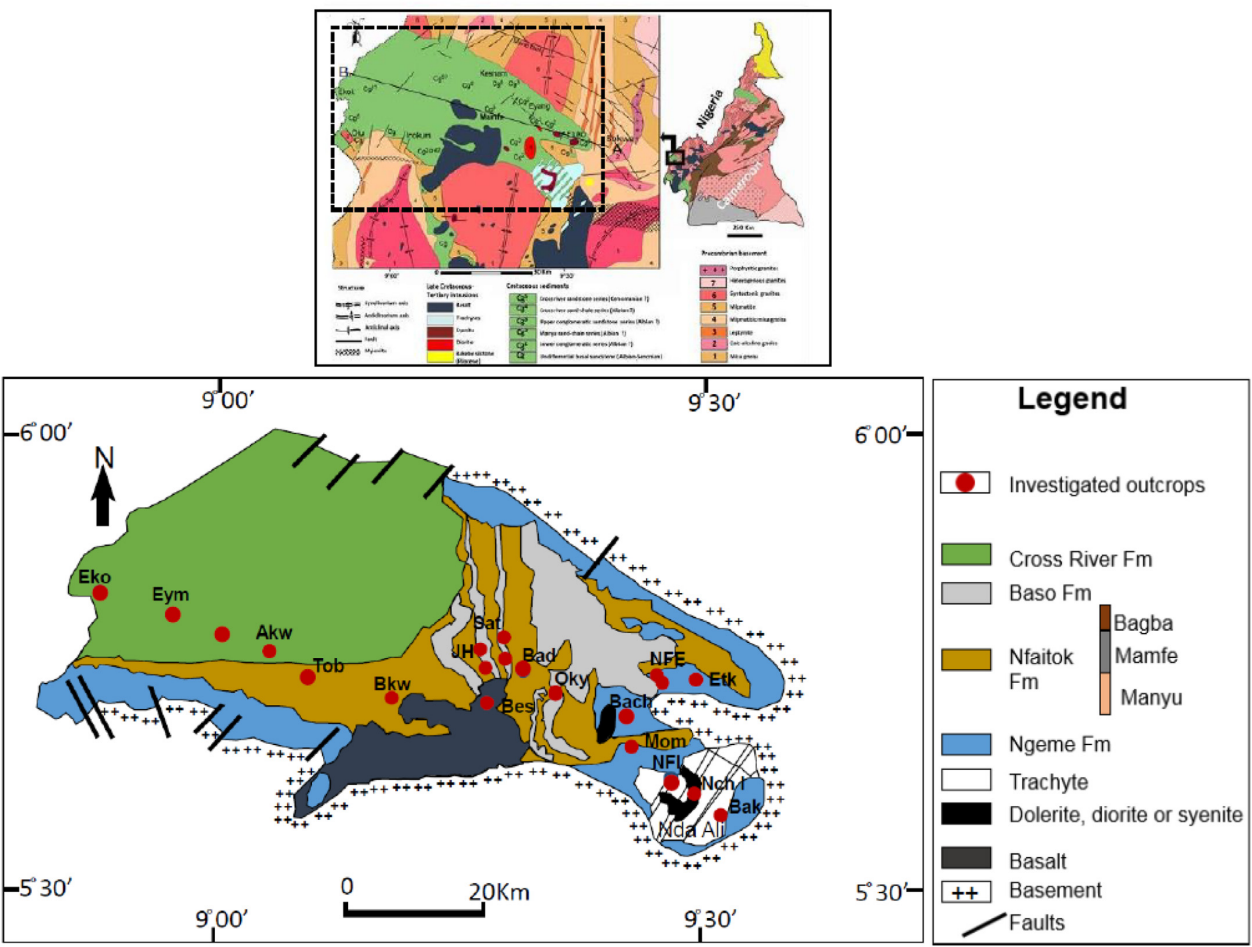
Geological map of the Mamfe Basin (above) and detailed map of the study area after Eyong (2003), displaying the position of the investigated outcrops.

The sampling site are mainly made up of road trenches and river banks (Figure 2). The choice of sample sites was based on accessibility, whereas the selection of samples was based on fresh and unweathered lithologies. A total of sixteen stratigraphic sections have been measured, described and sampled for subsequent analyses. Analyses include optical microscopy, scanning electron microscopy (SEM) and cathodoluminescence (CL). From the collected samples, 45 thin sections were produced and studied under optical microscopy. Twenty-one thin sections have been analyzed using both CL and SEM at the department of earth science of the University of Geneva (Switzerland).

Regarding geochemical analyses, samples were sawed, crushed and powdered using an Agathe mill into 200 μm mesh. Following about 50 mg of powder were dried in the oven at 110 °C for 24 h to remove organic matter and crystal water from minerals. The loss on ignition (LOI) values was constrained in accordance with the mass difference of rock powder and also aliquot before and after heating at 1050 °C in a furnace to oxidize iron content. Subsequently, 1.5 g of calcined rock powder were mixed with 6 g of lithium tetraborate (Li_2_B4O_7_) and fused for 5 min in a platine crucible to obtain lithium tetraborate glass beads using Eagon 2. Whole rock major element abundances were determined on the lithium tetraborate glass beads using a Philips PW2400 XRF spectrometer at the Institute of Earth Sciences, University of Lausanne. Contents in major and minor elements were recalculated on an anhydrous base before interpretation. Major elements SiO_2_, TiO_2_, Al_2_O_3_, Fe_2_O_3_, MnO, MgO, CaO, Na_2_O, K_2_O and P_2_O_5_ were obtained from fused pellet discs by XRF spectrometry using PANALYTICAL Philips PW2400 spectrometer, while BHVO-1, NIM-N, NIM-G, and SY-2 standards were used for quality control.

Traces and rare earth elements (REEs) were obtained on fused lithium tetraborate glass discs by LA-ICP-MS Agilent 7700X connected to UP-193 FX ablation system at the Institute of Earth Sciences, University of Lausanne. Each analyzed sample from triplicates and standard curve were used to ensure analysis precision.

## 4 Results

### 4.1 Distribution of major elements

**Figure 3:**
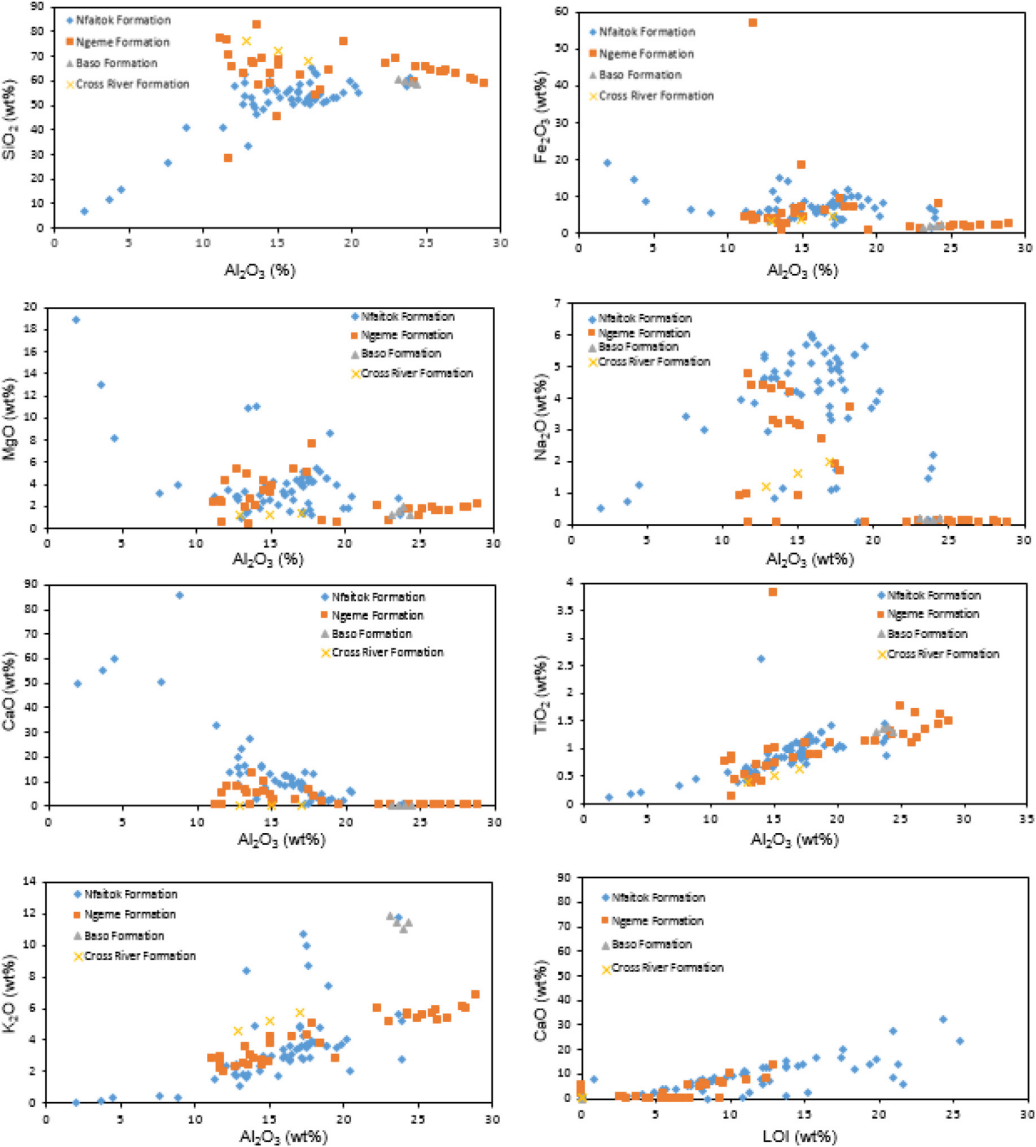
Diagram plots displaying the relationships between the selected major elements SiO2, Fe2O3, MgO, Na2O, TiO2, K2O against Al2O and CaO against LOI along with their different positive and/or negative correlation of the studied samples.

Major elements and major ratios of samples of 21 sites from the Mamfe Basin are presented in Table 1. Selected major oxides, including SiO2, Fe2O3, MgO, Na2O, TiO2, K2O against Al2O and CaO against loss on ignition (LOI) are shown in Figure 3 to highlight the relationships between these major elements. Among other major oxides, only SiO_2_, K_2_O, and TiO_2_ exhibit a positive linear correlation with Al_2_O_3_.

**Table 1:** Summary major elements concentration and major ratios of clastic rock from Mamfe Basin.

	Ngeme Formation			Nfaitok Formation			Baso Formation			Cross River Formation		
	Max.	Min.	Mean	Max.	Min.	Mean	Max	Min.	Mean.	Max.	Min.	Mean
SiO_2_	82.66	27.75	63.41	65.13	6.97	51.64	60.55	58.61	59.56	76.06	67.99	72.03
TiO_2_	3.80	0.13	1.04	2.64	0.12	0.89	1.39	1.29	1.34	0.64	0.39	0.52
Al_2_O_3_	28.93	11.22	18.79	23.90	1.94	15.72	24.38	23.09	23.74	17.04	12.89	14.96
Fe_2_O_3_	56.59	0.36	5.57	19.02	1.43	7.38	2.53	1.53	1.96	4.61	3.23	3.92
MnO	2.91	0	0.16	2.91	0.01	0.34	0.01	0.01	0.01	0.01	0.01	0.01
MgO	7.50	0.29	2.54	18.95	0.95	3.89	1.99	1.20	1.51	1.34	1.18	1.2636541
CaO	13.25	0	2.56	85.63	0.05	13.07	0.09	0.05	0.06	0.45	0.35	0.40
Na_2_O	4.72	0.03	1.64	6.00	0.07	3.89	0.19	0.12	0.16	2.01	1.21	1.61
K_2_O	6.82	1.95	4.07	11.79	0.07	3.52	11.86	11.07	11.45	5.78	4.58	5.18
P_2_O_5_	1.62	0.02	0.19	13.51	0.11	0.59	0.19	0.15	0.17	0.11	0.09	0.10
Al_2_O_3_/TiO_2_	31.85	20.17	23.49	20.97	13.78	16.34	18.93	17.22	17.78	32.97	26.53	29.75
SiO_2_/Al_2_O_3_	4.94	3.11	4.04	4.92	2.83	3.29	2.62	2.40	2.51	5.90	3.99	4.95
K_2_O/Na_2_O	2.946	1.06	1.73	0.28	0.17	0.21	91.42	57.6	74.31	3.78	2.88	3.33
K_2_O/Al_2_O_3_	0.278	0.25	0.26	0.29	0.17	0.21	0.51	0.46	0.48	0.36	0.34	0.35
CIA	61.99	47.96	55.41	70.26	2.10	40.26	66.81	64.29	65.75	63.61	62.75	63.18
CIW	1.446	1.20	1.34	84.09	2.10	44.77	0.69	0.69	0.69	0.87	0.85	0.86
ICV	76.26	55.43	66.14	45.5	0.64	3.62	100.29	99.94	100.13	84.21	81.54	82.872
PIA	83.63	39.24	59.44	80.19	2.03	39.56	222.89	199.42	208.32	105.27	93.82	99.55

### 4.2 Distribution of REEs

The concentrations of REEs (in ppm) and REEs elemental ratios of samples from the whole 21 sites studied in the Mamfe Basin are displayed in Table 2.

**Table 2:** REE element concentration (ppm) and REE elemental ratio from the Mamfe Basin.

Samples	La	Ce	Pr	Nd	Sm	Eu	Gd	Tb	Dy	Ho	Er	Tm	Yb	Lu	REE	LREE	HREE	L/H	(La/yb)N	(Ce/Ce∗)N
NFE1	80.50	163.00	17.03	63.30	11.50	2.29	9.18	1.30	8.40	1.63	4.74	0.68	4.53	0.65	368.73	323.83	44.90	35.28	12.06	0.70
NFE2	52.37	105.67	11.70	44.10	8.59	1.85	7.44	1.18	7.69	1.57	4.53	0.65	4.28	0.60	252.21	213.83	38.38	28.75	8.32	0.73
NFE3	50.86	104.41	11.79	45.56	8.97	1.82	7.67	1.09	6.88	1.32	3.54	0.50	3.31	0.48	248.21	212.62	35.59	27.73	10.44	0.74
NFE4	67.37	130.00	14.37	54.57	9.48	1.79	7.15	0.91	5.37	0.98	2.59	0.38	2.45	0.35	297.76	266.30	31.46	37.23	18.68	0.71
NFE5	87.40	177.00	17.60	65.27	10.40	1.99	7.66	0.92	5.11	0.93	2.55	0.36	2.43	0.36	379.97	347.27	32.70	45.35	24.40	0.68
NFE6	24.80	52.50	6.28	25.20	5.28	1.13	4.71	0.74	4.89	0.99	2.88	0.41	2.85	0.41	133.06	108.78	24.28	23.10	5.92	0.79
NFE7	71.27	153.33	14.97	57.00	9.83	1.93	7.56	0.98	6.09	1.12	3.20	0.45	3.19	0.46	331.38	296.57	34.81	39.25	15.16	0.70
NFE9	39.70	82.70	9.05	34.37	6.71	1.45	5.92	0.89	5.89	1.20	3.55	0.53	3.63	0.53	196.12	165.82	30.30	27.99	7.42	0.74
NFE10	69.20	138.00	14.77	56.37	9.57	1.84	7.20	0.90	5.38	0.95	2.57	0.36	2.41	0.36	309.86	278.33	31.52	38.68	19.53	0.71
NFE11	70.20	142.67	14.90	55.23	9.42	1.78	7.22	0.97	5.93	1.10	3.07	0.45	3.12	0.46	316.52	283.00	33.52	39.18	15.27	0.70
NFE12	83.37	160.67	16.93	60.13	9.13	1.53	6.00	0.74	4.43	0.83	2.35	0.35	2.47	0.36	349.29	321.10	28.19	53.52	22.96	0.68
NFE13	51.47	102.00	11.10	42.07	7.58	1.56	6.10	0.86	5.52	1.09	3.13	0.45	3.07	0.44	236.43	206.63	29.80	33.89	11.40	0.71
NFE14	68.23	137.67	14.97	55.47	9.66	1.92	7.33	0.97	5.81	1.09	3.11	0.44	2.90	0.44	310.00	276.33	33.66	37.72	15.98	0.72
NFE15	57.18	117.44	12.70	47.41	8.75	1.58	6.78	0.94	5.70	1.05	2.91	0.42	2.77	0.42	266.06	234.73	31.33	34.60	14.01	0.72
NFE16	62.10	132.00	13.83	52.43	9.66	1.91	7.61	1.05	6.65	1.29	3.52	0.52	3.51	0.51	296.59	260.37	36.22	34.23	12.03	0.73
NFE18	79.03	156.33	16.67	62.80	10.73	2.00	8.22	1.04	6.42	1.19	3.28	0.45	3.03	0.42	351.62	314.83	36.79	38.32	17.72	0.70
NFE19	46.93	94.13	10.13	40.23	7.42	1.43	6.00	0.79	4.80	0.85	2.33	0.32	2.11	0.30	170.86	191.43	26.36	31.89	15.09	0.71
NFE20	60.40	125.00	13.27	49.87	8.90	1.66	6.72	0.88	5.34	0.99	2.71	0.39	2.64	0.37	279.13	248.53	30.60	36.98	15.54	0.72
NFE 21	79.87	147.33	16.43	60.17	10.13	1.75	6.96	0.86	4.98	0.87	2.42	0.33	2.23	0.31	334.63	303.80	30.83	43.65	24.37	0.69
NFE22	83.31	154.98	17.23	63.00	10.35	1.78	7.47	0.91	5.16	0.92	2.50	0.35	2.30	0.34	350.58	318.52	32.06	42.64	24.57	0.69
NFE23	76.80	147.67	16.20	61.30	10.80	1.99	8.14	1.05	6.50	1.19	3.33	0.46	3.16	0.44	339.02	301.97	37.05	37.11	16.53	0.70
NFE24	70.90	131.67	13.97	48.87	7.91	1.42	5.55	0.71	4.08	0.74	1.99	0.29	1.96	0.29	290.33	265.40	24.93	47.82	24.62	0.67
NFE25	23.24	54.33	5.90	23.13	5.05	1.24	4.65	0.72	4.73	0.96	2.72	0.39	2.64	0.38	130.09	106.60	23.49	22.94	5.99	0.79
NFE26	18.10	42.23	4.53	18.93	4.52	1.17	4.34	0.65	4.40	0.87	2.49	0.35	2.44	0.33	105.36	83.80	21.56	19.31	5.04	0.78
SAT1	115.33	198.67	21.93	82.23	12.77	2.11	9.22	1.11	6.29	1.08	2.99	0.40	2.77	0.39	457.30	418.17	39.13	45.34	28.28	0.65
SAT3	62.27	111.33	11.57	40.47	6.53	1.07	4.50	0.56	3.40	0.61	1.70	0.25	1.75	0.24	246.24	225.63	20.61	50.14	24.13	0.64
SAT4	61.27	123.33	13.00	47.83	7.82	1.24	5.37	0.73	4.55	0.83	2.34	0.35	2.39	0.34	271.41	245.43	25.97	45.70	17.41	0.70
SAT5	76.37	147.67	15.77	56.33	8.89	1.43	5.99	0.76	4.28	0.72	2.08	0.30	1.86	0.26	322.71	296.13	26.57	49.41	27.94	0.69
SAT6	54.61	104.82	10.96	38.82	6.37	1.01	4.43	0.58	3.52	0.66	1.85	0.27	1.81	0.28	229.98	209.22	20.76	47.28	20.54	0.68
SAT 7	93.03	179.67	17.83	61.03	8.36	1.27	4.85	0.58	3.30	0.56	1.57	0.22	1.57	0.22	374.07	351.57	22.50	72.49	40.17	0.66
SAT8	44.07	86.67	9.32	33.20	5.52	0.95	4.07	0.55	3.43	0.65	1.97	0.29	2.05	0.29	193.02	173.25	19.76	42.60	14.60	0.70
SAT9	50.80	105.00	13.17	55.97	9.52	1.50	7.81	0.95	5.55	0.96	2.50	0.32	2.07	0.28	256.38	224.93	31.45	28.80	16.67	0.80
SAT10	52.73	101.33	10.77	39.17	6.49	1.07	4.54	0.59	3.71	0.72	2.03	0.29	2.14	0.29	225.87	204.00	21.87	44.93	16.74	0.69
SAT11	58.73	102.27	12.00	48.23	7.33	1.07	5.71	0.72	4.29	0.77	2.18	0.30	2.17	0.29	246.07	221.23	24.84	38.72	18.36	0.69
SAT12	60.17	115.00	11.97	43.53	7.21	1.26	5.12	0.66	4.04	0.74	2.09	0.30	2.17	0.31	254.57	230.67	23.90	45.02	18.86	0.67
SAT13	56.03	111.33	12.07	45.00	7.81	1.41	5.96	0.79	4.83	0.91	2.53	0.37	2.47	0.34	251.85	224.43	27.42	37.66	15.43	0.71
SAT14	50.53	95.03	9.91	35.97	6.08	1.10	4.40	0.58	3.60	0.65	1.82	0.25	1.81	0.26	211.99	191.44	20.55	43.48	18.97	0.67
SAT15	55.00	115.00	12.43	46.43	7.96	1.31	5.65	0.74	4.52	0.82	2.28	0.34	2.23	0.31	255.02	228.87	26.15	40.53	16.75	0.73
SAT 16	139.67	257.33	28.63	104.33	14.47	1.98	7.93	0.87	4.69	0.79	2.21	0.33	2.30	0.33	565.86	529.97	35.89	66.80	41.31	0.69
SAT17	49.87	100.00	11.10	42.23	7.70	1.43	6.24	0.85	5.32	1.01	2.90	0.40	2.73	0.39	232.15	203.20	28.95	32.58	12.41	0.72
SAT18	83.50	167.33	18.07	66.43	11.23	1.94	8.06	1.02	6.00	1.06	2.86	0.39	2.57	0.36	370.83	335.33	35.49	41.60	22.04	0.71
NFI1	110.33	194.00	20.30	73.07	12.47	2.29	10.00	1.28	7.39	1.23	3.03	0.36	2.27	0.30	438.33	397.70	40.63	39.78	33.07	0.64
NFI2	116.33	221.00	22.67	82.20	13.33	2.35	10.01	1.27	7.20	1.26	3.25	0.42	2.63	0.35	484.28	442.20	42.08	44.18	30.01	0.66
NFI3	133.00	256.00	27.87	100.33	15.10	2.66	8.71	0.94	4.83	0.86	2.32	0.35	2.29	0.32	555.59	517.20	38.39	59.36	39.40	0.70
NFI4	120.61	240.42	25.03	89.94	14.19	2.49	8.96	1.05	5.77	1.05	2.96	0.42	2.85	0.42	516.16	475.99	40.16	53.14	28.71	0.69
NFI5	29.33	72.09	7.27	27.21	4.88	0.97	3.84	0.52	2.77	0.49	1.28	0.19	1.29	0.20	152.33	135.90	16.43	35.38	15.41	0.78
NFI6	73.48	158.40	17.70	68.37	13.14	2.15	10.60	1.37	7.81	1.38	3.57	0.52	3.55	0.54	362.57	317.95	44.62	30.01	14.07	0.76
NFI7	172.00	342.67	37.87	136.33	22.43	3.96	14.93	1.82	9.75	1.63	4.41	0.60	4.17	0.58	753.16	688.87	64.29	46.13	28.00	0.72
NFI8	114.33	211.00	23.67	85.10	13.20	2.39	9.08	1.08	6.01	1.05	2.78	0.39	2.56	0.38	473.03	434.10	38.93	47.83	30.38	0.69
NFI9	139.67	255.00	27.50	101.23	17.33	3.59	14.90	1.83	10.23	1.65	3.91	0.48	2.93	0.42	580.68	523.40	57.28	35.13	32.38	0.67
NFI10	73.57	137.67	16.27	62.27	11.30	2.20	8.49	1.10	6.45	1.13	3.17	0.45	3.13	0.44	327.63	289.77	37.87	34.12	15.97	0.72
NFI11	121.30	230.19	26.05	95.63	15.86	2.69	10.72	1.31	7.28	1.31	3.61	0.54	3.51	0.50	520.50	473.17	47.33	44.14	23.49	0.71
NFI12	55.30	115.67	12.67	48.83	8.88	1.98	6.76	0.91	5.93	1.10	3.27	0.48	3.34	0.49	265.60	232.47	33.13	34.37	11.25	0.74
NFI14	13.73	30.43	3.73	15.50	3.50	0.84	3.25	0.49	3.32	0.63	1.85	0.28	1.89	0.28	79.73	63.39	16.34	19.53	4.94	0.82
NFI15	99.20	198.33	22.13	84.73	14.40	3.15	10.77	1.36	8.50	1.64	4.87	0.71	4.69	0.72	455.21	404.40	50.81	37.56	14.36	0.73
NFI16	63.53	131.33	14.97	59.27	12.03	1.93	10.93	1.44	8.46	1.53	4.02	0.58	3.92	0.57	314.52	269.10	45.42	24.61	11.02	0.75
BAK1	127.67	240.33	26.63	97.20	17.53	3.01	14.47	2.03	12.60	2.34	6.27	0.87	5.67	0.83	557.45	491.83	65.62	34.00	15.30	0.70
BAk2	19.07	34.00	4.33	17.30	3.40	0.69	3.16	0.45	2.87	0.59	1.72	0.24	1.72	0.26	89.78	74.69	15.08	23.66	7.55	0.73
BAK 3	21.00	38.73	4.77	18.77	3.49	0.72	3.40	0.48	3.11	0.59	1.82	0.25	1.78	0.27	99.18	83.27	15.90	24.52	8.00	0.73
BAK4	17.80	32.89	4.12	15.86	3.16	0.62	2.86	0.42	2.62	0.55	1.56	0.23	1.57	0.24	84.51	70.68	13.83	24.71	7.70	0.74
MOM1	41.39	84.01	9.58	39.09	8.34	1.93	7.49	1.06	6.40	1.19	3.24	0.44	2.90	0.43	207.49	174.07	33.42	23.25	9.70	0.74
MOM2	50.24	101.37	11.25	44.53	8.92	2.03	8.10	1.15	7.31	1.43	3.86	0.54	3.67	0.54	244.94	207.39	37.55	25.60	9.29	0.73
MOM3	42.12	89.10	9.97	39.42	8.04	1.61	6.68	0.92	5.62	1.06	2.86	0.43	2.91	0.43	211.19	180.62	30.57	27.04	9.82	0.75
MOM5	57.87	116.95	13.26	53.18	11.17	2.34	9.89	1.48	9.23	1.81	4.89	0.67	4.78	0.67	288.20	241.26	46.94	24.40	8.22	0.74
mom7	12.17	24.66	3.01	12.63	3.19	0.92	3.72	0.62	4.32	0.86	2.49	0.36	2.60	0.38	71.95	52.48	19.47	14.11	3.17	0.77
BES1	5.76	6.22	5.92	5.70	5.77	5.33	5.89	5.39	5.77	5.67	5.84	5.24	6.17	5.29	79.97	23.60	56.36	4.01	0.63	1.45
BES2	104.00	195.00	22.80	84.95	15.50	2.63	11.90	1.52	8.78	1.61	4.01	0.55	3.32	0.47	457.01	406.75	50.26	34.18	21.31	0.72
BES4	133.14	241.11	26.75	98.65	16.23	2.84	11.87	1.46	8.61	1.56	4.23	0.59	4.07	0.59	551.69	499.65	52.04	42.11	22.22	0.68
BES5	35.21	60.41	6.25	22.76	3.75	0.68	3.02	0.38	2.37	0.47	1.26	0.18	1.18	0.18	138.09	124.62	13.47	41.23	20.21	0.62
BES7	0.57	1.03	0.11	0.41	0.07	0.01	0.05	0.01	0.04	0.01	0.02	0.00	0.02	0.00	2.36	2.12	0.24	39.71	17.39	0.66
BES8	68.28	134.20	14.44	53.21	8.98	1.70	6.90	0.87	5.12	0.94	2.52	0.36	2.24	0.32	300.07	270.12	29.95	39.16	20.75	0.70
BES9	97.63	177.70	18.83	69.47	12.01	2.09	9.08	1.15	6.50	1.21	3.17	0.45	3.04	0.42	402.74	363.62	39.11	40.04	21.79	0.66
BES10	67.14	116.05	12.53	45.19	7.60	1.34	5.85	0.76	4.37	0.83	2.18	0.30	1.96	0.27	266.36	240.90	25.45	41.16	23.31	0.65
JH1	118.00	199.33	19.53	70.93	11.77	2.11	8.75	1.12	6.55	1.22	3.25	0.45	2.97	0.44	446.42	407.80	38.62	46.62	26.99	0.59
JH3B	125.33	233.67	25.17	89.77	14.43	2.66	9.38	1.11	6.08	1.04	2.81	0.40	2.66	0.38	514.89	473.93	40.95	50.51	32.05	0.68
JH3B	106.25	198.76	20.87	76.02	11.86	1.94	8.29	1.01	5.79	1.06	2.91	0.42	2.82	0.43	438.42	401.89	36.53	48.50	25.58	0.67
JH4	27.65	28.52	13.33	19.50	8.04	2.49	5.58	1.17	4.33	1.26	2.88	0.53	2.95	0.52	118.74	88.99	29.75	15.95	6.37	1.10
JH5	42.44	78.77	8.83	33.75	6.09	1.20	4.85	0.64	3.94	0.74	2.10	0.28	1.97	0.28	185.87	163.78	22.09	33.78	14.61	0.69
JH8	52.86	111.61	14.10	61.79	13.12	3.86	11.19	1.43	8.03	1.27	3.05	0.36	2.11	0.27	285.06	240.37	44.69	21.48	17.01	0.81
JH12	130.55	252.22	27.31	98.66	16.53	2.94	11.21	1.36	7.61	1.34	3.50	0.48	3.37	0.47	557.55	508.74	48.81	45.38	26.32	0.70
ETK2	30.60	62.70	6.93	28.17	5.71	1.33	4.98	0.74	4.50	0.83	2.26	0.31	2.05	0.29	151.40	128.40	23.00	25.77	10.14	0.73
ETK3	47.90	87.80	8.86	32.57	5.92	1.35	4.46	0.64	3.89	0.73	2.07	0.30	2.07	0.28	198.82	177.12	21.70	39.74	15.74	0.64
ETOK5	68.96	129.60	13.33	49.22	8.58	1.73	6.34	0.78	4.23	0.73	1.90	0.26	1.65	0.23	287.55	261.11	26.44	41.19	28.31	0.66
ETK5	151.69	291.70	30.18	110.62	18.32	3.75	13.45	1.59	8.91	1.56	4.17	0.60	4.02	0.57	641.12	584.19	56.93	43.44	25.62	0.67
AKW1	407.67	822.43	90.09	358.55	77.15	21.70	75.70	10.69	64.58	12.76	35.25	5.42	35.47	5.28	2022.75	1678.75	344.00	22.18	7.81	0.72
AKW3	152.11	203.02	43.61	110.38	26.67	5.26	17.02	2.42	12.27	2.48	6.99	1.12	8.32	1.32	592.99	509.12	83.88	29.91	12.42	0.85
MCH1	85.14	158.88	17.58	64.30	10.89	2.07	8.29	1.02	5.97	1.08	2.92	0.40	2.74	0.38	361.66	325.89	35.77	39.29	21.14	0.69
MCH2	54.94	104.21	11.63	45.12	8.35	1.80	6.97	0.95	5.66	1.04	2.83	0.40	2.60	0.37	246.88	215.91	30.97	30.97	14.33	0.70
MCH3	58.71	106.04	12.04	46.66	9.24	1.98	8.41	1.18	7.39	1.40	3.75	0.55	3.42	0.49	261.26	223.45	37.81	26.56	11.65	0.69
Mchdy	59.50	124.44	15.30	65.74	12.77	4.51	11.26	1.40	7.86	1.38	3.38	0.43	2.55	0.34	310.85	264.97	45.88	23.54	15.83	0.79
BKP1	76.45	158.73	16.39	63.13	10.97	2.15	9.36	1.30	7.94	1.51	4.09	0.58	4.00	0.57	357.16	314.70	42.46	33.62	12.98	0.71
BKP2	60.10	128.57	13.10	49.25	8.86	1.73	7.38	1.00	5.99	1.15	3.14	0.48	3.14	0.45	284.32	251.02	33.30	34.00	13.02	0.72
BKP3	74.46	153.37	15.77	59.28	10.43	1.95	8.42	1.11	6.81	1.33	3.52	0.53	3.46	0.52	340.96	302.88	38.08	35.96	14.60	0.70
BKW1	60.24	125.93	13.77	54.48	10.94	2.28	9.75	1.36	8.59	1.68	4.79	0.69	4.68	0.70	299.87	254.41	45.46	26.08	8.75	0.74
BKW3	92.90	167.12	22.74	95.38	19.81	5.06	17.96	2.48	14.73	2.63	6.86	0.89	5.65	0.78	455.00	378.14	76.86	21.05	11.17	0.77
OKY2	155.60	315.12	35.64	135.08	23.85	4.57	17.02	2.12	11.40	2.00	5.07	0.68	4.28	0.57	713.01	641.44	71.57	37.69	24.68	0.74
BAD	125.11	251.33	27.26	103.23	18.38	3.53	13.24	1.62	9.14	1.63	4.28	0.59	3.91	0.57	563.81	506.93	56.88	38.28	21.74	0.72
TOB1	147.72	241.23	35.94	116.42	28.68	7.77	43.76	8.98	65.83	14.13	36.77	4.82	25.60	3.43	781.08	541.31	239.77	12.37	3.92	0.77

NASC 32.00 73.00 7.90 33.00 5.70 1.24 5.20 0.85 5.80 1.04 3.40 0.50 3.10 0.48 173.21.

NASC refers to the North American shale from Haskin et al., 1968.

Chrondites 0.237 0.613 0.0928 0.457 0.148 0.0567 0.199 0.0361 0.246 0.0546 0.16 0.0247 0.161 0.0246 (McDonough and Sun, 1995).

### 4.3 Distribution of trace elements

Trace elements concentrations (in ppm) of samples from the total 21 sites of the Mamfe Basin are given in Table 3.

**Table 3:** Trace element concentration (ppm) from the Mamfe Basin.

Samples	Sc	V	Cr	Mn	Ni	Cu	Zn	Ga	Rb	Sr	Zr	Nb	Cs	Ba	Hf	Ta	Pb	Th	U
NFE1	20.60	115.20	73.50	1592.20	26.57	104.67	27.73	20.70	81.87	520.33	136.67	16.33	2.74	643.00	3.57	1.08	7.24	19.30	2.04
NFE2	18.37	118.10	69.70	1384.60	20.50	84.80	21.60	21.50	77.43	454.33	155.67	15.43	2.50	553.67	4.14	1.15	8.44	22.37	2.38
NFE3	17.02	86.10	72.10	907.30	41.84	38.93	34.54	21.90	97.55	565.07	136.73	15.66	4.11	930.94	3.78	1.11	10.71	27.56	2.19
NFE4	15.00	111.90	101.30	488.10	69.03	31.50	51.67	28.50	121.00	323.67	115.33	20.17	6.23	816.00	3.25	1.38	9.10	23.03	2.43
NFE5	15.00	188.70	129.10	322.50	87.57	38.03	64.20	27.60	133.67	172.00	154.00	25.87	6.64	765.67	4.11	1.89	7.93	21.90	4.30
NFE6	14.77	113.10	58.20	1126.60	19.33	32.50	13.80	19.20	72.57	250.00	238.67	14.67	1.21	613.00	6.15	1.10	9.83	15.93	2.60
NFE 7	17.27	108.50	95.40	823.30	52.90	58.40	56.57	26.30	112.00	473.67	128.33	19.17	5.60	803.67	3.61	1.30	9.40	18.07	3.07
NFE9	16.87	57.00	56.90	1658.30	14.40	14.40	22.10	16.60	59.03	310.67	289.67	11.20	1.24	440.33	7.82	0.96	6.46	10.60	3.06
NFE10	16.07	137.50	125.30	429.90	77.90	13.87	78.57	29.90	123.33	199.33	146.33	22.53	6.50	746.33	4.29	1.57	7.40	18.33	4.27
NFE11	17.97	139.40	113.20	740.60	67.27	42.63	67.63	27.30	116.67	344.33	142.33	20.37	6.08	772.00	4.11	1.41	7.51	22.73	5.50
NFE12	17.10	144.50	82.20	622.00	55.70	47.80	51.60	21.50	168.00	799.67	66.83	16.13	8.77	1150.00	1.76	1.10	88.67	19.00	8.16
NFE13	21.57	95.20	77.80	1113.10	40.07	103.33	52.73	21.20	100.00	585.67	163.00	16.63	4.81	720.67	4.43	1.14	7.95	14.20	3.52
NFE14	22.00	123.20	102.30	1114.20	52.57	108.67	54.83	26.00	121.33	394.33	127.67	20.67	5.92	818.33	3.45	1.39	7.93	20.07	3.68
NFE15	16.54	158.20	115.50	497.40	74.18	4.02	84.63	28.20	129.92	292.05	118.48	20.53	6.91	766.69	3.34	1.37	7.58	23.62	6.40
NFE16	22.83	123.00	92.20	1275.50	41.07	44.67	44.13	22.20	107.67	526.00	129.33	18.93	4.90	805.67	3.50	1.24	8.00	20.73	3.22
NFE18	21.10	121.60	94.80	1106.60	42.77	117.00	61.00	23.10	113.67	430.00	105.33	19.53	5.05	784.00	2.95	1.32	8.93	16.00	5.71
NFE19	14.70	154.50	126.40	478.20	80.77	50.57	67.00	30.80	140.00	253.33	96.00	21.13	7.49	755.00	2.75	1.42	7.33	23.93	4.43
NFE20	38.77	150.70	94.30	650.80	67.57	35.93	61.17	26.80	141.33	457.00	75.67	17.60	7.15	850.33	2.05	1.16	9.97	22.20	5.57
NFE 21	18.67	166.90	91.30	769.40	54.00	49.57	49.80	24.90	159.33	421.67	67.43	16.43	7.96	844.33	1.65	1.01	31.67	17.97	6.87
NFE22	18.95	100.00	70.00	500.00	54.69	46.34	55.39	36.40	164.15	431.37	70.08	16.83	8.23	865.06	1.71	1.08	32.78	18.79	7.32
NFE23	23.37	101.80	78.90	1501.60	29.93	119.67	139.67	19.50	105.33	546.00	82.37	17.30	3.91	791.00	2.22	1.09	18.67	21.50	4.47
NFE24	16.73	139.50	108.60	541.20	66.37	17.53	58.20	28.90	182.33	326.33	67.57	19.20	9.89	811.33	1.89	1.27	8.86	16.33	4.21
NFE25	13.08	68.80	49.70	2183.90	15.09	69.53	11.97	16.20	54.03	400.57	133.08	9.62	0.75	580.37	3.50	0.82	8.98	9.16	3.62
NFE26	10.60	100.40	22.60	1191.40	6.05	38.67	13.37	13.20	44.13	415.33	153.67	5.50	0.57	984.00	3.45	0.54	10.18	6.65	1.68
SAT1	14.37	117.80	50.20	4656.10	39.77	65.63	187.33	24.00	115.00	405.00	151.00	20.87	6.64	600.33	4.28	1.41	18.57	25.40	6.96
SAT2	1.17	26.10	4.10	8332.80	41.95	9.39	433.00	1.40	1.32	17.20	20.30	9.77	2.07	25.25	0.48	0.20	0.70	0.13	0.25
SAT3	12.27	100.90	45.20	597.00	29.17	35.47	25.00	28.70	115.00	335.00	142.00	19.57	5.71	443.33	4.00	1.55	16.57	20.60	6.47
SAT 4	12.50	57.90	29.10	1309.20	16.40	32.23	27.67	15.00	60.97	446.33	126.67	11.30	2.81	295.00	3.47	0.89	10.53	12.13	4.27
SAT5	12.30	110.30	47.40	803.70	27.03	32.97	28.03	26.20	104.33	376.33	154.67	19.43	4.95	393.33	4.54	1.48	18.47	20.13	6.98
SAT6	12.21	56.60	32.90	1386.00	18.61	21.05	30.90	20.40	101.74	311.84	222.78	14.26	4.78	432.63	5.94	1.19	16.00	14.23	4.58
SAT 7	12.63	102.20	53.90	338.20	45.97	32.17	44.70	34.60	185.00	308.33	150.00	23.87	10.60	517.33	4.28	1.81	20.57	24.27	7.05
SAT8	8.57	44.60	25.90	2110.80	11.83	31.33	22.93	15.40	64.03	267.00	243.00	12.37	2.95	315.33	6.53	1.14	13.27	10.93	4.23
SAT9	6.69	50.90	17.30	2849.20	14.07	14.30	7.52	4.30	10.37	401.67	52.10	4.85	0.27	166.67	1.40	0.36	3.80	5.82	3.53
SAT10	9.62	52.60	30.30	1383.40	12.30	40.00	22.67	18.20	76.07	304.67	279.67	14.40	3.40	345.67	7.54	1.20	13.87	14.50	4.89
SAT11	13.57	68.00	36.90	1154.60	15.87	21.13	24.10	23.50	119.33	238.67	216.33	14.63	4.96	401.00	5.76	1.13	13.13	13.47	3.72
SAT12	13.20	83.00	46.70	1124.30	18.63	30.40	31.37	24.20	121.33	298.00	212.33	17.53	5.84	522.00	5.76	1.34	15.50	15.57	4.79
SAT13	9.00	67.40	29.30	2086.40	13.87	22.67	39.80	12.70	49.27	443.33	111.67	10.02	2.53	307.00	3.10	0.81	12.10	10.77	5.12
SAT14	10.23	91.90	39.10	1831.60	16.47	21.90	25.27	21.00	89.10	304.33	154.67	15.17	4.33	357.00	4.20	1.13	18.23	13.67	5.67
SAT15	9.68	76.50	33.40	2335.50	16.37	30.63	21.73	14.40	58.43	356.67	157.33	12.27	2.64	358.67	4.27	1.00	18.77	11.37	5.73
SAT16	17.50	120.50	65.70	107.00	26.57	38.97	45.07	36.50	236.00	207.67	149.67	27.07	10.00	629.67	4.22	1.94	15.40	29.70	8.87
NFI1	20.37	144.80	82.00	22.30	12.73	17.53	16.53	35.70	223.00	61.60	88.60	24.17	20.00	482.67	2.65	1.72	73.07	32.20	9.56
NFI2	18.63	135.40	78.80	21.10	18.33	12.63	18.77	35.60	197.33	71.57	124.33	25.27	15.77	487.67	3.63	1.75	39.93	30.80	9.22
NFI3	26.30	140.10	118.30	27.80	20.17	15.73	26.43	39.30	264.00	22.20	93.83	26.33	16.97	514.00	2.57	1.77	13.07	28.23	5.09
NFI4	26.50	140.60	122.70	28.00	19.23	13.64	21.21	38.90	224.12	28.27	144.70	25.23	15.27	484.53	3.87	1.69	21.19	25.94	3.76
NFI5	9.08	31.40	28.00	16.90	11.70	9.44	15.25	15.60	110.55	14.10	199.57	11.31	3.27	468.89	5.31	0.99	7.63	8.36	2.33
NFI6	12.75	75.10	83.30	8.50	18.30	9.45	9.22	21.80	93.16	39.61	652.94	21.84	3.46	334.22	17.08	1.71	12.89	21.27	4.95
NFI7	21.00	146.80	82.40	31.30	8.68	36.77	12.13	36.90	231.67	68.97	113.33	27.10	18.40	650.67	3.40	1.91	39.50	31.70	9.47
NFI8	23.57	166.50	101.20	20.30	16.70	23.13	12.93	34.70	212.00	34.23	107.67	23.77	16.93	334.00	3.07	1.59	30.50	29.13	6.36
NFI9	19.67	148.00	97.90	20.10	17.00	24.93	12.93	34.60	209.00	79.03	109.33	23.97	16.40	500.67	3.28	1.67	31.17	31.67	9.21
NFI10	25.70	205.30	142.10	25.20	13.30	23.57	13.23	39.50	213.67	57.67	97.40	24.23	16.93	401.33	2.75	1.58	21.27	30.43	6.96
NFI11	25.57	159.40	107.10	26.40	15.36	20.06	12.76	37.30	202.80	57.68	110.44	25.04	16.29	425.34	3.13	1.71	32.60	31.11	6.97
NFI12	25.03	183.70	169.10	15.20	18.23	17.07	12.37	38.40	167.33	26.47	361.33	27.67	8.88	444.67	9.75	1.83	19.87	15.37	4.75
NFI14	23.50	207.20	192.70	0.00	10.37	47.97	96.13	21.20	71.20	14.17	146.67	10.93	4.32	167.00	3.86	0.76	62.13	12.10	6.93
NFI15	27.97	154.70	128.40	18.60	20.60	10.33	16.23	37.40	199.67	31.07	270.33	28.63	12.63	388.00	7.36	2.03	53.63	25.77	7.49
NFI16	19.10	111.60	89.60	11.00	9.66	3.20	11.73	29.10	139.33	42.43	682.67	19.67	6.45	437.67	18.50	1.47	8.41	20.77	6.21
BAK1	9.64	23.20	6.60	1305.40	7.44	5.79	142.00	32.90	108.67	225.33	773.67	163.67	2.09	1490.00	17.60	9.29	16.27	19.77	4.66
BAk2	15.97	77.90	78.40	311.20	32.60	5.01	61.33	19.30	118.00	102.33	223.33	15.57	6.72	605.67	5.99	1.25	14.40	7.99	2.34
BAK 3	15.50	53.10	50.90	305.70	28.20	5.29	61.50	14.30	105.67	110.00	183.00	14.97	5.00	651.00	5.12	1.70	14.57	9.07	2.22
BAK4	14.29	52.70	50.10	304.70	22.63	3.26	45.84	14.50	87.26	93.98	160.85	12.75	4.11	546.12	4.46	1.42	11.26	7.75	1.88
MOM1	14.02	60.60	61.50	1587.40	30.18	75.03	62.73	16.20	72.87	209.03	276.72	11.56	1.87	507.92	7.47	0.79	8.58	10.20	2.70
MOM2	10.53	27.80	26.60	1499.50	11.34	5.35	7.62	12.30	76.35	233.37	291.00	7.05	1.61	755.16	7.70	0.52	7.49	12.20	2.54
MOM3	9.91	155.60	1.70	3197.60	7.12	13.39	3.79	29.30	86.75	225.09	285.34	7.54	1.94	472.91	7.49	0.56	7.99	12.69	2.53
MOM5	13.32	22.60	28.30	1575.00	13.93	44.57	19.41	9.40	48.85	230.21	352.72	8.06	1.17	538.70	9.24	0.56	8.78	11.46	3.20
mom7	13.37	16.40	9.60	1279.00	6.26	5.50	173.27	8.60	61.46	318.42	66.67	2.59	1.24	859.27	1.86	0.18	12.86	2.88	0.80
MOM8	120.40	31.20	29.50	1965.70	125.30	74.01	859.17	10.30	474.24	1827.59	2798.13	80.79	10.80	4627.60	74.47	5.83	70.45	81.08	21.68
BES1	6.46	64.70	55.80	108.40	5.96	5.84	6.16	23.20	5.13	12.36	5.85	6.08	6.31	6.12	5.51	5.69	6.28	5.97	5.86
BES2	21.45	243.30	80.80	271.40	55.15	80.70	94.65	34.10	131.00	509.00	99.95	21.90	8.67	625.50	3.03	1.48	19.50	25.45	11.60
BES4	22.75	733.70	83.70	496.90	61.80	130.22	5790.90	31.40	101.54	249.02	154.60	23.40	7.83	450.53	4.32	1.62	33.90	30.28	35.55
BES5	3.98	89.50	9.80	2594.60	14.39	24.89	34.49	3.30	4.48	398.13	28.15	3.58	0.17	74.76	0.72	0.25	1.41	4.53	8.25
BES7	0.08	64.40	10.90	3057.70	0.25	0.59	0.20	3.80	0.21	18.35	0.55	0.07	0.01	2.59	0.02	0.01	0.16	0.09	0.09
BES8	15.07	262.60	62.30	18900.30	51.52	106.41	22.01	19.90	74.90	883.39	78.23	15.32	3.78	587.39	2.24	1.07	24.53	18.89	9.18
BES9	18.38	161.10	74.10	10072.80	71.53	118.28	48.12	22.10	77.17	582.37	77.43	18.72	4.65	480.11	2.16	1.26	21.72	23.59	6.87
BES10	9.85	81.60	17.50	3492.30	33.69	82.36	24.01	5.70	16.15	1999.62	39.45	9.75	0.79	698.47	1.11	0.69	5.39	13.63	11.20
JH1	12.90	336.80	49.60	505.10	78.43	169.00	34.63	18.30	62.47	1093.33	73.23	15.07	4.97	611.33	1.96	1.02	133.00	19.17	17.90
JH4	7.59	45.80	14.80	6373.70	10.04	20.54	12.22	15.00	7.58	104.70	15.07	13.13	2.71	13.85	5.61	1.63	28.03	12.55	7.39
JH5	6.54	22.20	15.10	2455.40	6.38	19.10	18.79	12.30	73.44	414.87	252.22	9.59	2.36	2004.03	6.73	0.83	15.08	10.40	2.54
JH8	19.59	146.20	243.00	661.80	260.13	52.59	172.06	22.10	43.43	552.40	450.21	61.20	0.71	630.68	9.96	3.68	2.38	4.61	1.32
JH12	21.48	108.00	112.60	44.90	19.32	8.11	26.35	35.60	182.52	300.48	211.69	30.56	6.25	1102.85	5.92	2.39	18.47	37.45	8.59
ETK2	11.77	27.40	16.90	930.30	9.51	9.21	18.10	14.60	85.60	491.00	256.33	10.57	2.30	1360.00	6.92	0.80	13.50	8.89	2.52
ETK3	10.60	27.40	16.90	930.30	20.60	20.37	50.07	14.60	121.00	529.67	237.67	18.60	5.59	1680.00	6.21	1.38	18.60	19.30	6.89
ETOK5	8.61	129.90	63.70	720.80	21.25	35.56	47.88	26.20	108.79	152.80	81.39	10.01	10.60	524.77	1.51	0.70	26.14	15.58	4.33
ETK5	26.40	91.60	53.30	499.50	59.64	54.37	133.16	25.90	311.97	830.33	440.96	35.38	22.40	2629.16	11.53	2.55	29.37	42.53	14.02
AKW1	153.24	40.30	38.80	79.60	228.42	136.14	445.03	15.10	2267.87	5646.14	3071.26	145.05	70.25	18433.21	81.88	11.10	311.47	140.67	30.03
AKW3	34.01	46.00	37.30	92.80	54.08	11.42	102.87	22.70	268.08	383.05	294.85	57.00	27.34	344.98	34.15	5.89	56.66	81.45	15.55
MCH1	21.67	133.60	102.20	826.20	63.03	40.19	132.17	24.90	174.81	538.62	97.75	21.48	11.45	1137.89	2.64	1.43	15.10	23.69	7.66
MCH2	14.41	65.00	52.30	2567.10	29.06	37.02	237.03	16.30	105.52	976.55	128.77	14.56	5.95	1082.54	3.42	0.95	19.03	13.41	8.00
MCH3	16.90	82.30	81.40	1209.10	41.61	112.55	96.50	18.40	91.42	650.08	157.55	16.74	5.17	1018.30	4.13	1.15	16.04	15.10	4.04
Mchdy	17.66	205.70	0.30	23212.30	4.28	11.22	285.19	21.00	90.12	867.38	264.84	68.87	0.58	456.19	6.13	4.40	3.53	4.73	1.19
BKP1	12.60	59.10	57.90	587.70	22.00	16.12	27.72	21.00	160.20	309.86	329.35	21.95	3.28	950.38	8.98	1.82	15.36	24.24	6.28
BKP2	10.38	42.20	42.10	4506.00	17.70	15.81	33.13	13.80	126.05	233.84	211.80	17.46	2.78	771.67	5.78	1.48	9.81	18.25	4.76
BKP3	13.05	62.00	57.30	337.10	29.00	6.84	25.83	22.40	173.33	300.36	319.34	21.03	4.31	1002.74	8.74	1.75	13.40	24.11	6.03
BKW1	19.26	112.70	108.50	283.70	52.86	7.50	101.99	25.70	171.98	376.95	400.20	18.51	8.59	1505.73	10.88	1.37	20.64	15.88	5.26
BKW2	3692.38	147.10	147.10	366.10	11378.15	1097.43	20411.92	32.90	30578.10	41320.66	8099.70	2989.34	1439.48	169120.09	237.79	195.80	2651.49	2725.90	484.81
BKW3	33.19	202.00	153.30	345.70	59.56	181.53	133.38	34.20	235.68	128.99	83.65	26.30	12.56	459.95	2.30	1.78	43.60	22.71	4.26
OKY2	20.87	91.90	104.60	46.90	23.63	13.21	34.96	39.40	207.85	328.94	280.35	30.33	4.21	1033.38	7.87	2.40	17.37	35.93	9.77
BAD	18.89	93.40	99.50	50.60	21.56	16.53	44.19	39.90	200.06	434.81	247.75	31.99	8.17	1168.49	7.12	2.66	22.55	34.98	12.27
TOB1	33.99	136.90	68.30	393.30	69.07	16.37	208.86	35.70	281.05	402.44	145.62	48.58	23.25	362.65	8.84	3.98	44.40	41.73	15.43

UC 11.00 60.00 355.00 600.00 20.00 25.00 71.00 17.00 112.00 350.00 190.00 25.00 4.90 50.00 3.90 2.20 20.00 10.70 2.80.

UC refers to average upper crust and is from Taylor and McLennan, 1885.

### 4.4 Statistics

The summary statistics results of REEs concentrations of samples from the Ngeme and Nfaitok formations in the Mamfe Basin are listed in Table 4.

**Table 4:** Summary statistics REE concentration (ppm) of the Mamfe Basin.

	Baso Formation (n = 2)			Cross River Formation (n = 3)			Nfaitok Formation (n = 60)			Nfaitok Formation (n = 39)		
	Mean	Max	Min	Mean	Max	Min	Mean	Max	Min	Mean	Max	Min
ƩREE (ppm)	1307.87	2022.75	592.99	685.97	781.08	563.81	1192.53	55845.35	2.36	374.41	1762.16	71.95
LREE	1093.93	1678.75	509.12	1689.69	641.44	506.93	1014.19	46717.77	2.12	328.80	1427.34	52.48
HREE	213.94	344.00	83.88	368.22	239.77	56.88	179.10	9127.58	0.24	45.62	334.82	13.83
L/H	26.04	29.91	22.18	88.34	38.28	12.37	37.82	72.49	4.01	33.28	59.36	14.11
Dy_N_/Sm_N_	0.39	0.50	0.28	0.66	1.38	0.29	0.38	0.60	0.20	0.39	0.81	0.19
La_N_/Yb_N_	10.11	12.42	7.81	16.78	24.68	3.92	17.46	41.31	0.63	17.98	39.40	3.17
Gd_N_/Yb_N_	1.69	1.73	1.66	2.45	3.21	1.38	2.14	4.29	0.77	2.36	4.11	1.16
(La/La∗)_N_	0.99	1.28	0.69	1.12	1.23	0.96	1.22	1.55	0.15	1.24	1.41	1.07
(Ce/Ce∗)_N_	0.78	0.85	0.72	0.74	0.77	0.72	0.72	1.45	0.59	0.72	0.82	0.64
(Eu/Eu∗)_N_	0.69	0.78	0.61	0.58	0.59	0.54	0.57	0.94	0.44	0.62	1.04	0.46
(Pr/Pr∗)_N_	1.28	1.64	0.91	1.02	1.19	0.92	1.04	5.64	0.86	0.93	0.97	0.88

Table 5 presents the correlation coefficient results showing relationships between various REEs concentrations from Ngeme and Nfaitok formations.

**Table 5:** Correlation coefficient of the relationships between various REE element concentrations of Ngeme and Nfaitok formations.

Ngeme Formation (N = 33)																				
	Sc	V	Cr	Mn	Ni	Cu	Zn	Ga	Rb	Sr	Zr	Nb	Cs	Ba	Hf	Ta	Pb	Th	U	ƩREE
Sc	1																			
V	0.01	1																		
Cr	0.10	0.68	1																	
Mn	-0.01	0.20	-0.39	1																
Ni	0.81	-0.22	-0.04	-0.10	1															
Cu	0.36	-0.03	0.10	-0.04	0.57	1														
Zn	0.83	-0.22	-0.28	0.31	0.78	0.39	1													
Ga	-0.03	0.75	0.61	-0.17	-0.26	-0.20	-0.38	1												
Rb	0.80	0.23	0.25	-0.17	0.65	0.22	0.50	0.41	1											
Sr	0.64	-0.31	-0.43	0.38	0.75	0.50	0.88	-0.47	0.38	1										
Zr	0.85	-0.30	-0.23	0.04	0.73	0.27	0.83	-0.26	0.55	0.67	1									
Nb	0.31	-0.06	-0.21	0.27	0.19	-0.02	0.44	0.20	0.30	0.31	0.49	1								
Cs	0.28	0.51	0.51	-0.32	0.20	0.08	-0.08	0.72	0.76	-0.11	-0.08	0.01	1							
Ba	0.75	-0.43	-0.36	0.00	0.83	0.40	0.82	-0.34	0.60	0.86	0.81	0.42	0.08	1						
Hf	0.87	-0.30	-0.21	0.03	0.74	0.27	0.83	-0.26	0.56	0.67	1.00	0.46	-0.07	0.81	1					
Ta	0.40	-0.08	-0.22	0.28	0.28	-0.01	0.51	0.16	0.37	0.36	0.57	0.99	0.03	0.49	0.54	1				
Pb	0.58	0.23	0.39	-0.24	0.35	0.24	0.34	0.29	0.61	0.16	0.33	0.15	0.55	0.30	0.34	0.19	1			
Th	0.84	0.13	0.18	-0.21	0.69	0.32	0.56	0.31	0.94	0.45	0.67	0.35	0.65	0.67	0.68	0.42	0.66	1		
U	0.78	0.11	0.18	-0.20	0.71	0.40	0.59	0.19	0.86	0.54	0.63	0.29	0.60	0.70	0.64	0.35	0.73	0.93	1	
ƩREE	0.87	0.04	0.01	-0.03	0.68	0.33	0.69	0.21	0.88	0.55	0.77	0.50	0.48	0.71	0.78	0.56	0.56	0.94	0.85	1
Nfaitok Formation (n = 62)																				

	Sc	V	Cr	Mn	Ni	Cu	Zn	Ga	Rb	Sr	Zr	Nb	Cs	Ba	Hf	Ta	Pb	Th	U	ƩREE
Sc	1																			
V	0.04	1																		
Cr	0.25	0.33	1																	
Mn	-0.07	0.00	-0.29	1																
Ni	1.0	0.04	0.26	-0.07	1															
Cu	.96	0.19	0.30	-0.02	.96	1														
Zn	.96	0.26	0.25	-0.08	.96	.94	1													
Ga	0.18	0.04	0.63	-0.38	0.18	0.22	0.20	1												
Rb	1.0	0.04	0.25	-0.07	1.0	.96	.96	0.18	1											
Sr	1.0	0.04	0.24	-0.06	1.0	.96	.96	0.16	1.0	1										
Zr	1.0	0.03	0.26	-0.09	1.0	.95	.96	0.18	1.0	.99	1									
Nb	1.0	0.04	0.26	-0.07	1.0	.96	.96	0.18	1.0	1.0	1.0	1								
Cs	1.0	0.04	1.00	-0.07	1.0	.96	.96	0.18	1.0	1.0	1.0	1.0	1							
Ba	1.0	0.04	0.24	-0.07	1.0	.96	.96	0.17	1.0	1.0	1.0	1.0	1.0	1						
Hf	1.0	0.02	0.25	-0.08	1.0	.95	.96	0.18	1.0	.99	1.0	1.0	1.0	1.0	1					
Ta	1.0	0.04	0.25	-0.08	1.0	.96	.96	0.18	1.0	1.0	1.0	1.0	1.0	1.0	1.0	1				
Pb	1.0	0.06	0.24	-0.07	1.0	.96	.96	0.18	1.0	1.0	.99	1.0	1.0	1.0	.99	1.0	1			
Th	1.0	0.04	0.25	-0.07	1.0	.96	.96	0.19	1.0	1.0	1.0	1.0	1.0	1.0	1.0	1.0	1.0	1		
U	1.0	0.10	0.24	-0.07	1.0	.96	.98	0.19	1.0	1.0	.99	1.0	1.0	1.0	.99	1.0	1.0	1.0	1	
ƩREE	1.0	0.04	0.24	-0.07	1.0	.96	.96	0.18	1.0	1.0	1.0	1.0	1.0	1.0	1.0	1.0	1.0	1.0	1.0	1

## 5 Interpretation

The chimical constituents of clastic sedimentary rocks is linked to those of its parental source. Therefore, the geochemistry of clastic rocks appears as a useful tool for reconstructing provenance, paleoweathering, as well as tectonic regime and paleoenvironmental conditions prevailing during deposition. For this purpose, major, trace and REEs and their elemental ratios can be considered as sensitive recorders, and are commonly used to understand the sedimentation condition of basin infill (Bathia, 1983; Roser and Korsch, 1986; Mc Lennan and Taylor, 1991; Mc Lennan et al., 1993; Condie, 1993; Nesbit et al., 1996; Fedo et al., 1997; 2002; Singh, 2013; Kanhaiya et al., 2018).

### 5.1 Elemental geochemistry and mineralogic composition

The chemical alteration and hydraulic degradation of various mineral phases during sediment flow are susceptible to change eroded material. But this product always keeps details on the primitive igneous activities (Johnson, 1993). Fralick and Kronberg (1997) and Fralick (2003) showed that when samples from the same origin and containing non-mobile constituents have two of these non-mobile constituents traced together, a straight line of null intercord resulted. Generally, mobile constituents including Na, K, and large ion lithophile elements (LILEs) such as Cs, Rb, Ba, K, Pb, and light REEs (LREEs) decrease during the alteration process. In contrast, the static or non-mobile compounds such as Al_2_O_3_, TiO_2_ and REEs and high field strength elements (HFSEs) including Ta, Nb, Zr, Hf and Ti increased (Nesbit and Young 1982, 1984; McLennan et al., 1993). However, major elements distribution depends on the mineralogical constituent of the native rocks. This explain why the main compound of sandstones is SiO_2_ while shales mains constituents are K_2_O, Fe_2_O and TiO_2_ (Madhavaraju and Lee, 2009). In the present study, the SiO_2_ concentration is significant in the Ngeme and Cross River formations and varies from 27.75% to 82.66% (mean 63.40%) and from 67.99% to 76.05% (mean 72.02), respectively. It ranges from 6.87% to 65.13% (mean 51.63%) and 58.61%–60.55% (mean 59.56%) in the Nfaitok and Baso formations, respectively. Meanwhile, the Al_2_O_3_ content in the Ngeme and Cross River formations varies from 11.22 to 28.93 % (mean 18.78%) and from 12.88 to 17.03% (mean 14.96%), respectively. In contrast, it varies from 1.94 to 23.90% (mean 15.71) and from 23.09 to 24.37% (mean 23.74) in the Nfaitok and Baso formation, respectively. The content of K_2_O is higher than that of Na_2_O in the Ngeme, Nfaitok, Baso and Cross River formations (Table 1), suggesting the abundance of potassium-bearing compounds in the basin. Concentration of CaO is higher than that of MgO in the basin (Figures 5a and 5b).

**Figure 4:**
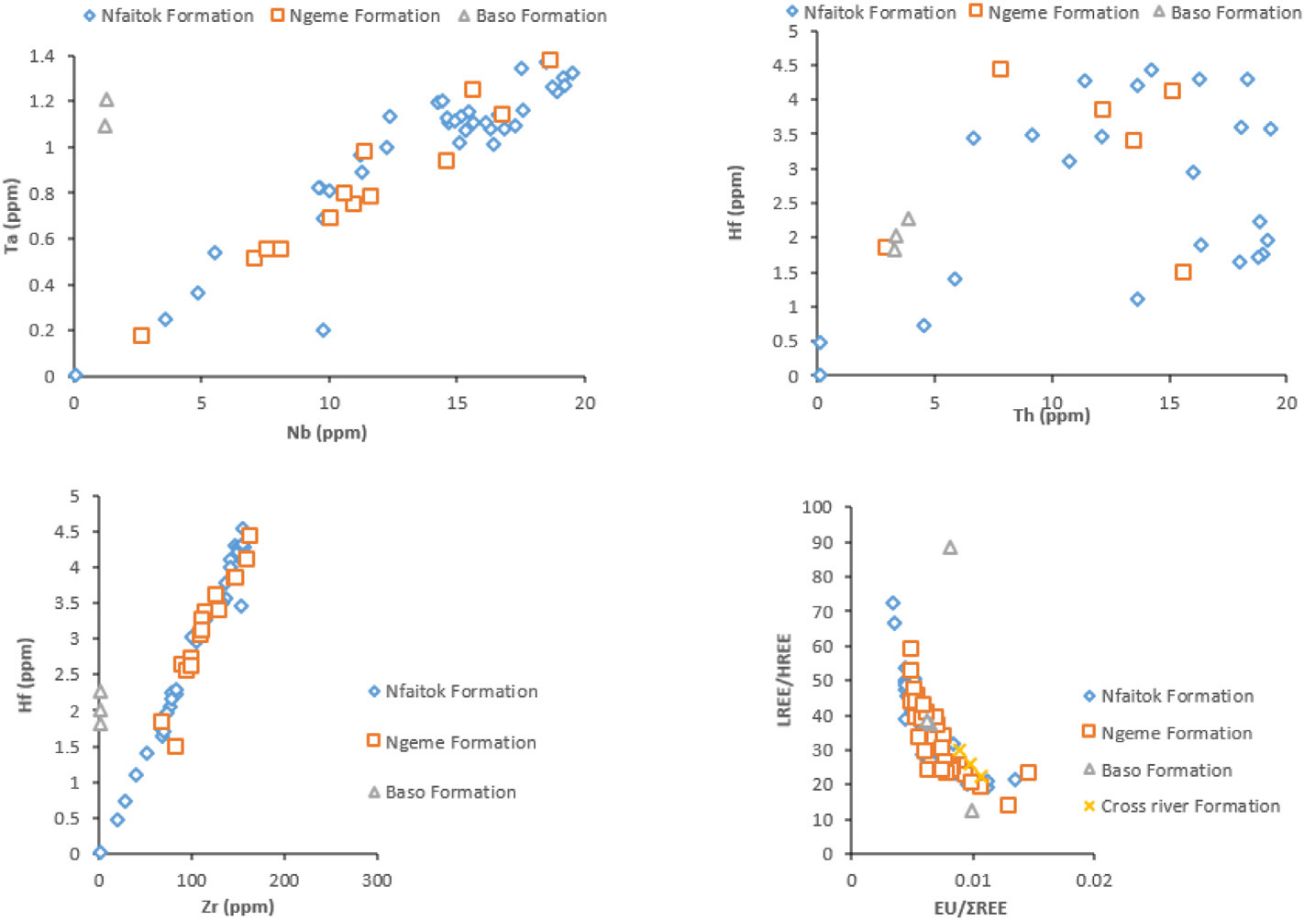
Bivariate plot diagrams displaying the relationships of traces elements and REEs of samples from different Formations of the Mamfe Basin.

Due to the static character of Al_2_O_3_ during the alteration process, its concentration serves as a significant standardisation component to establish a comparison between varied rocks (Bauluz et al., 2000). In the case of this study, only SiO_2_, K_2_O, and TiO_2_ exhibit a positive linear correlation with Al_2_O_3_ (Figure 3) indicative of their association with micas and clay rich components in the sediment of the basin (Das et al., 2006). The strong positive linear correlation between CaO and LOI (R^2^ = 0.80) implies that LOI and CaO are mostly from calcite than plagioclase, thus the enrichment of calcite in the sediment. A similar linear positive correlation exists with SiO_2_ and Al_2_O_3_ (R^2^ = 0.45), indicating that SiO_2_ is the most significant compound in the investigated samples includes detrital silicate as well as clay minerals. Whereas the positive linear correlation with K_2_O and Al_2_O_3_ (R^2^ = 0.32) reveals that clay and feldspars are the prime host minerals, TiO2 and Al_2_O_3_ (R^2^ = 0.21) suggest the phyllosilicates are enriched in Ti 4+ and its host mineral are from the same origin (Ross and Bustin, 2009). The strong negative correlation observed between Fe_2_O_3_, MgO, CaO, and Na_2_O indicate that these elements are mobile and not associate with Al_2_O_3_. Furthermore, the plotting of Nb vs Ta, Th vs Hf, Zr vs Hf and Eu/ΣREE vs ΣLREE/ΣHREE ratios in the Ngeme, Nfaitok, Baso and Cross River formations exhibit a similar trend in their distribution in Ngeme and Nfaitok formations (Figure 4) suggesting that the distribution of REEs in Ngeme and Nfaitok formations is controlled by the same factor and are from the same source rock.

**Figure 5:**
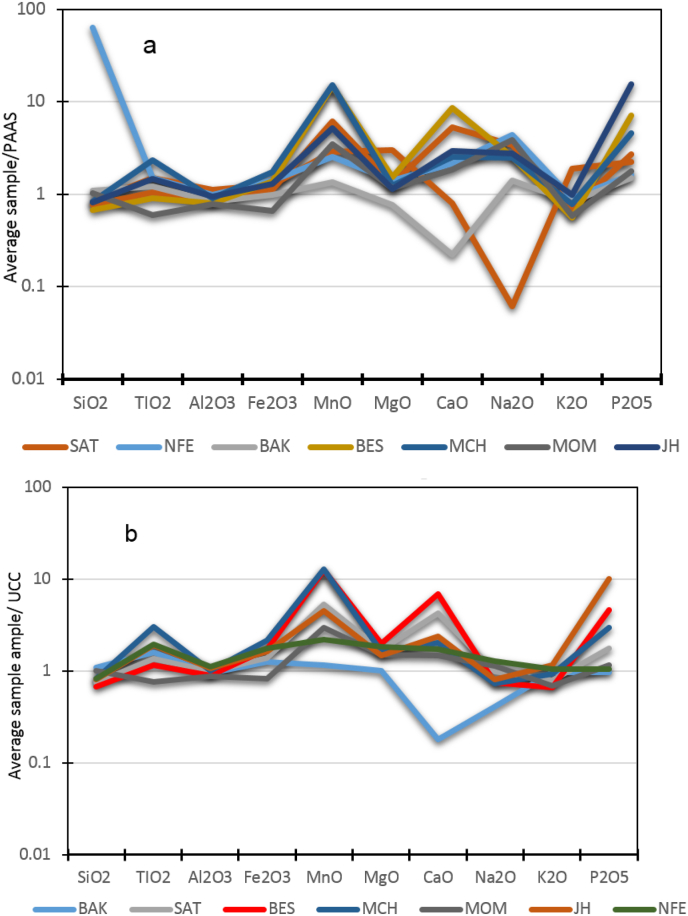
(a) Upper Continental Crust (UCC) normalized average major elements diagram. (b) Post-Archean Australian Shale (PAAS) normalized average major elements diagram.

The K_2_O/Al_2_O_3_ proportion in clay minerals oscillates from 0 to 0.3, while, in feldspars, it is from 0.3 to 0.9. In illite-rich sediments, the K_2_O/Al_2_O_3_ proportion ranges from 0.2 to 0.3 (Zhou et al., 2015), and K_2_O/Al_2_O_3_ ratio value is close to zero in sediment rich in kaolinite, smectite and vermiculite (Cox et al., 1995). The Mamfe Basin has the K_2_O/Al_2_O_3_ ratio (Table 1) ranges from 0.25 to 0.27 in Ngeme, 0.17 to 0.28 in Nfaitok, 0.46 to 0.51 in Baso and 0.33 to 0.35 in Cross River formations. These results reveal that sediments of Ngeme and Nfaitok formations are rich in clay minerals. In contrast, Baso and Cross River formations contain abundant plagioclase, indicating more alteration in Ngeme and Nfaitok formations. The occurrence of plagioclase in Baso and Cross River formations suggests short transport distance, rapid burial and less intensive weathering.

In this study, the SiO_2_/Al_2_O_3_ index value ranges from 3.11 to 4.94 in Ngeme; 2.82 to 4.91 in Nfaitok; 2.40 to 2.62 in Baso and 3.99 to 5.9 in Cross River formations indicate that clay minerals in Ngeme, Nfaitok and Baso formations include montmorillonite or smectite. Otherwise, the SiO_2_/Al_2_O_3_ ratio in the basin reflects weak silicification and significant aluminosilicate enrichment during diagenesis.

The Al_2_O_3_/TiO_2_ ratio varies from 20.17 to 31.85 in Ngeme; 13.7 to 20.96 in Nfaitok; 17.2 to 18.8 in Baso and 26.5 to 32.9 in Cross River formations suggesting a constant occurrence of phyllosilicate (micas) and clay minerals including Kaolinite in Ngeme, Nfaitok and Cross River formations. In contrast, illite and montmorillonite are enriched in Baso Formation.

According to Cullers (1994) and Fedo et al. (1995), during the diagenesis process, elements such as Ca, Mg, Na and few Fe and Sr are removed, while Si and K are enriched (Cullers, 1994). The K_2_O/Na_2_O ratio varies from 1.06 to 2.94 in Ngeme, 0.17 to 0.28 in Nfaitok, 57.6 to 91.41 in Baso and 2.88 to 3.77 in Cross River formations, implying a significant k-metasomatism process in Baso Formation. In contrast, the K_2_O/Na_2_O ratio implication is low in the other formations suggesting very limited mineral dissolution and replacement during diagenesis.

### 5.2 Geochemical classification from major elements

The SiO_2_/Al_2_O_3_ ratio allows discriminating quartz arenites with high content in Si to intermediate sandstone with high content in Al. It considered as good proxy for mineral maturity (Pettijohn et al., 1972). The Fe_2_O_3_/K_2_O proportion is thought to be a proxy for steady state of minerals. Accordingly, k-feldspar, muscovite and quartz represent the main steady state developing minerals at low temperature and pressure. The minerals k-feldspar and muscovite are rich in potassium, while quartz is poor in iron. In opposition, the low steady developing minerals is in lithic fragment with increasing in iron and magnesium. In general, steady minerals are poor in Fe_2_O_3_/K_2_O proportion. While few steady mineral assemblages commonly occur in the vicinity of the source origin with notable lithic debris and important proportion of Fe_2_O_3_/K_2_O (Herron, 1988).

**Figure 6:**
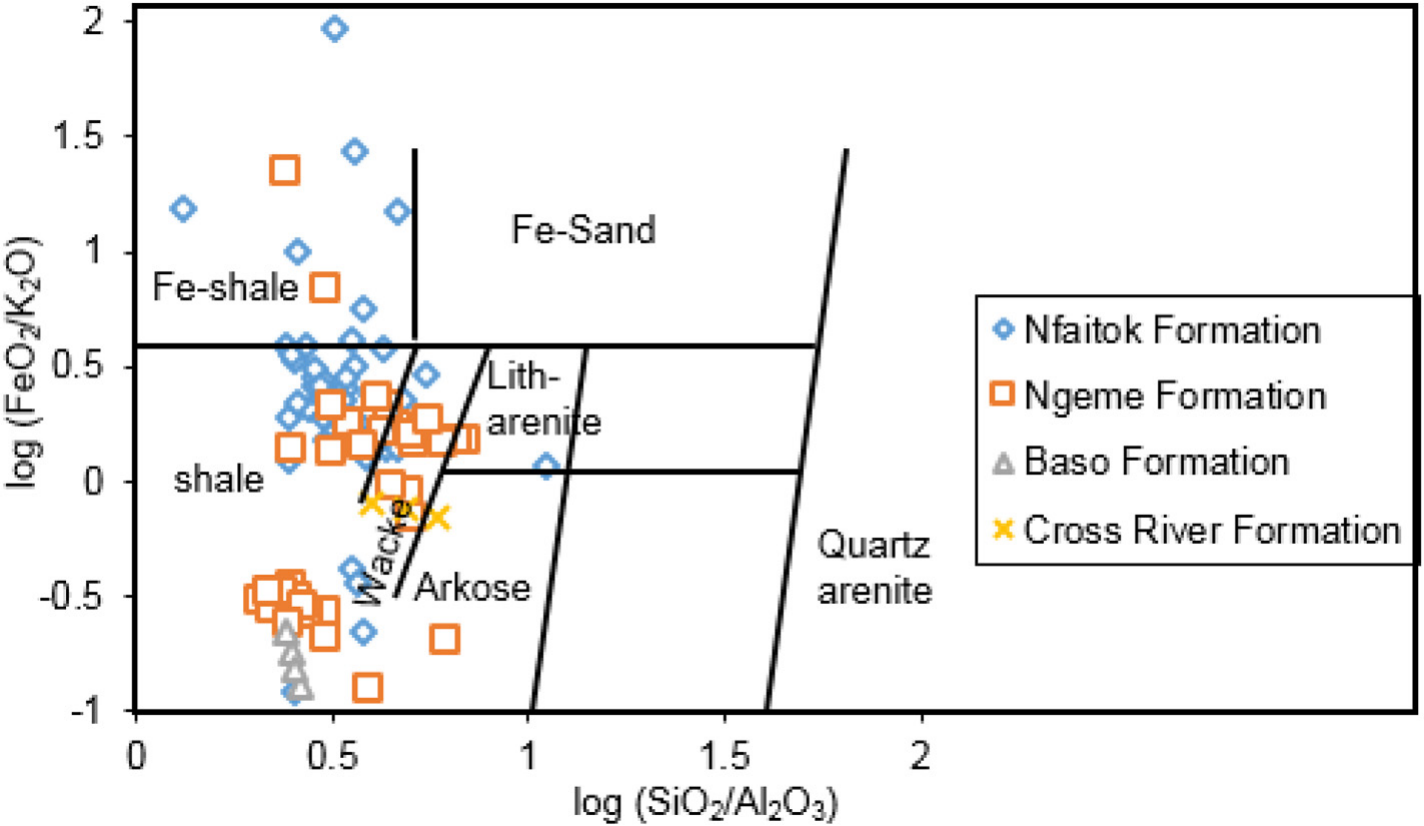
Geochemical classification of the clastic rocks of Mamfe samples using the log (SiO_2_/Al_2_O_3_)-log (Fe_2_O_3_/K_2_O) diagram after Herron (1988).

**Figure 7:**
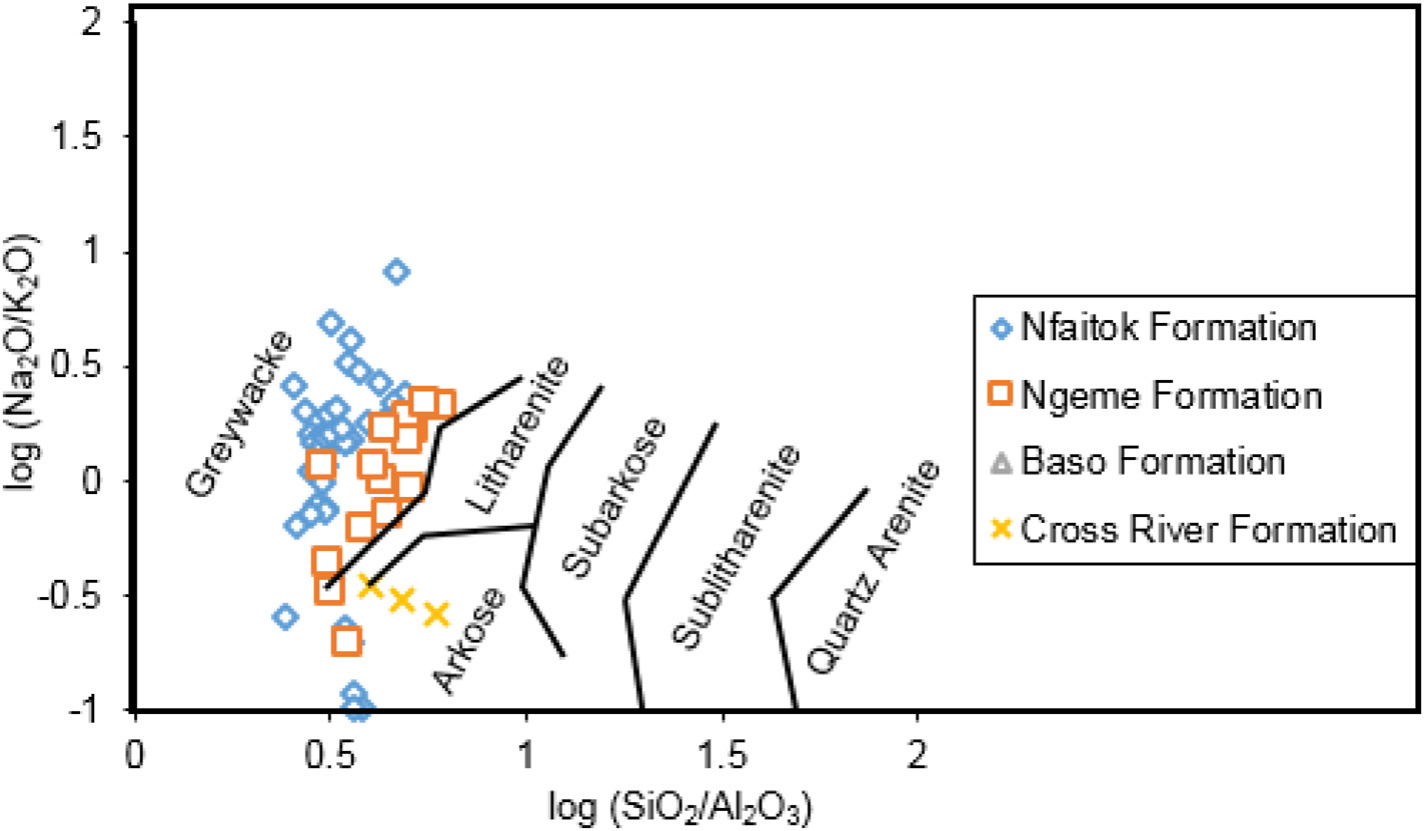
Geochemical classification of the clastic rock of the Mamfe samples using the log (SiO_2_/Al_2_O_3_)-log (Na_2_O/K_2_O) diagram after Pettijohn and Potter (1972).

Sediments from the MSB were assessed with the log ratios of SiO_2_/Al_2_O_3_ against Fe_2_O_3_/K_2_O to access the geochemical classification based on Herron (1988) (Figure 6). This plot shows that samples from Ngeme and Nfaitok formations are chemically composed of shale, Fe-shale, wacke whereas samples from the Cross River Formation consist of arkoses. Additionally, the Na_2_O/K_2_O ratios have distinguished greywacke to arkoses (Pettijohn, 1943, 1975; Middleton, 1960; Pettijohn et al., 1972). Moreover, to classify Mamfe clastic rock according to its chemical composition, the log proportion of Na_2_O/K_2_O traced with the log ratios of SiO_2_/Al_2_O_3_ after Pettijohn and Potter (1972) in Figure 7 show that sediments from Ngeme and Nfaitok formations consist mainly of greywackes, with arkoses in Cross River Formations.

### 5.3 Tectonic setting

**Figure 8:**
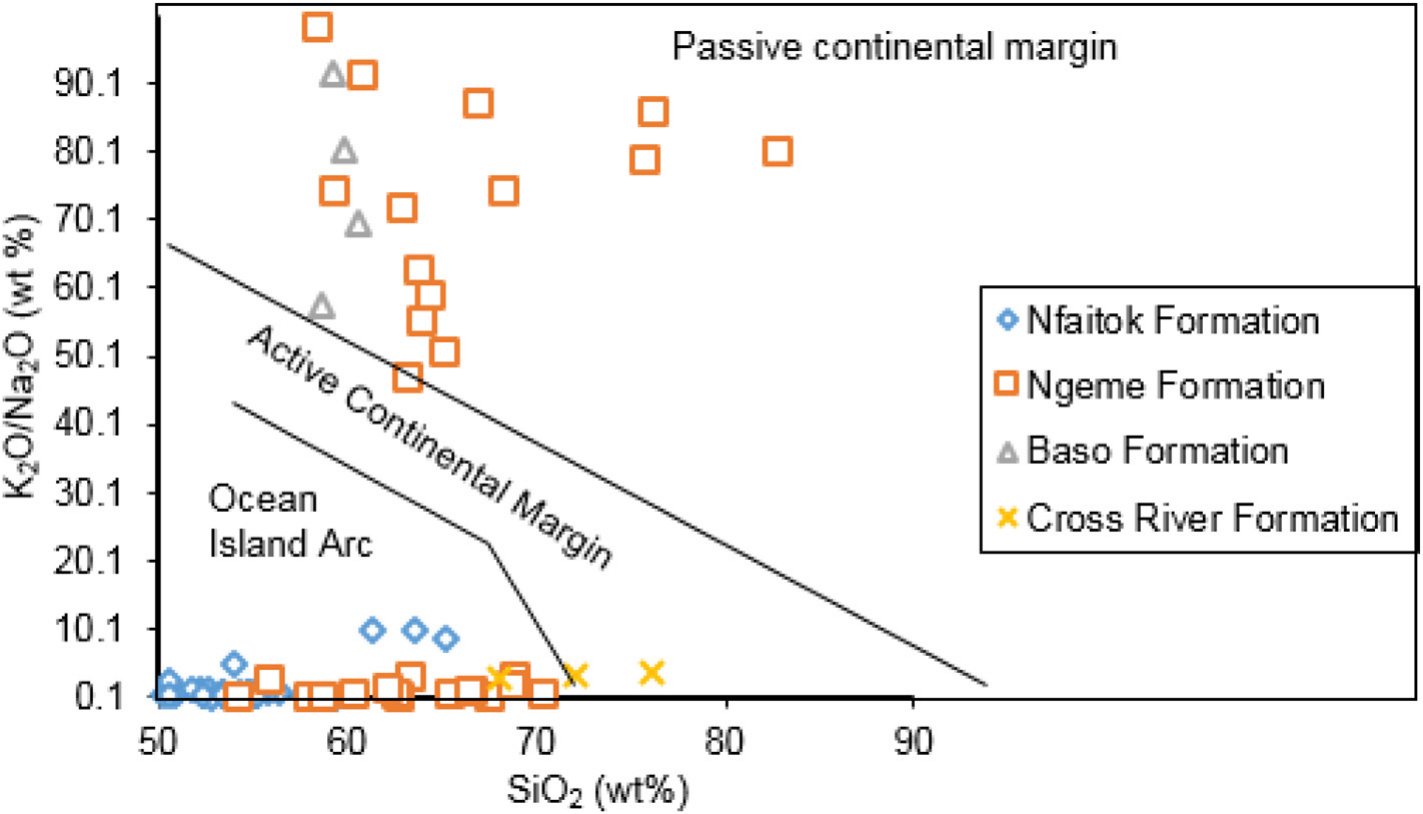
The position of the Mamfe samples within the SiO_2_ versus K_2_O/Na_2_O diagram to discriminate tectonic regimes using a provenance plot after Roser and Korsch (1986).

It is well established that the tectonic settings mainly control the chemical composition of clastic rocks. Hence, clastic rocks with various tectonic settings may be discriminated by geochemical signatures (Roser and Korsch, 1986). Generally, the K_2_O/Na_2_O ratios will become higher with the evolution of the island arc to continental island arc to active continental margin (Bhatia, 1983). The discrimination diagram of SiO_2_ vs K_2_O/Na_2_O ratios established by Roser and Korsch (1986) comprises passive margin, active margin and oceanic island arc. The SiO_2_ vs K_2_O/Na_2_O proportion of the investigated samples of the Mamfe Basin scattered in the area of passive continental margin for Ngeme and Baso formations. Whereas oceanic island Arc are found in Ngeme, Nfaitok and Cross River formations (Figure 8).

### 5.4 Provenance and source area composition

**Figure 9:**
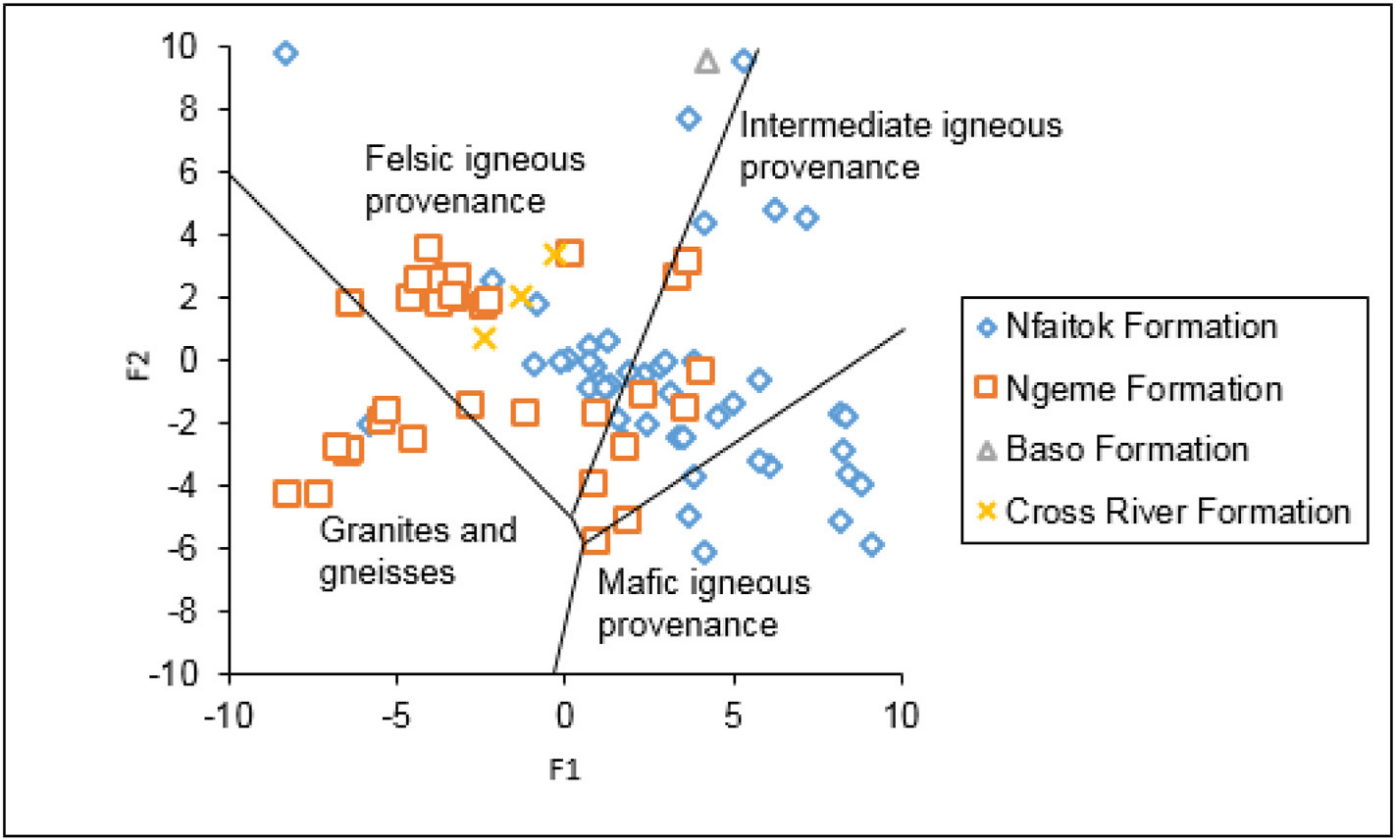
Major elements discriminant function diagram of the Mamfe sandstones and shales for sedimentary provenance after Roser and Korsch (1988).

**Figure 10:**
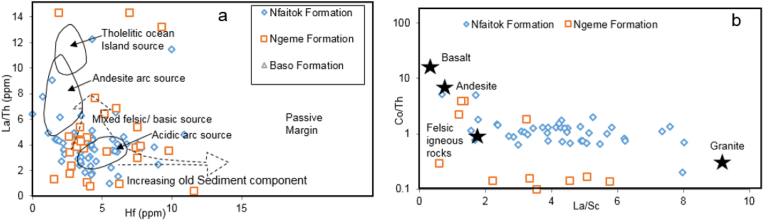
Source and compositional discrimination of the Ngeme, Nfaitok and Baso Formations based on Hf vs. La/Th and La/Sc vs. Co/Th (after Floyd and Leveridge, 1987).

The geochemistry of siliciclastic sediments is widely helpful to characterize its origin (Cullers et al., 1988; Taylor and McLennan, 1985; Condie et al., 1992). Numerous elemental plots using major, trace, and REEs have been proposed to determine sediments' provenance and source composition. The discriminant functions analysis using major elements Al_2_O_3_, TiO_2_, Fe_2_O_3_, MgO, CaO, Na_2_O and K_2_O from Roser and Korsch (1988) enable to discriminate among four source of sediment including mafic, intermediate, felsic, and recycled sedimentary rocks. These functions are calculated using the following formula: F1 = -1.773∗TiO_2_ + 0.607 × Al_2_O_3_ + 0.760 × Fe_2_O_3T_ - 1.500 × MgO +0.616 × CaO +0.509 × Na_2_O - 1.224 × K_2_O - 9.090 and F2 = 0.445 × TiO_2_ + 0.070 × Al_2_O_3_ - 0.250 × Fe_2_O_3T_ - 1.142 × MgO +0.438 × CaO +1.475 × Na_2_O + 1.426K_2_O - 6.861. Data and results of this study (Figure 9) suggest that sediments came largely from felsic and intermediate igneous with a small contribution of recycled quartzose sediment (mature polycyclic continental sedimentary rocks) and mafic igneous provenance. In addition, the discrimination diagrams of Hf vs La/Th (Figure 10a) according to Flyod and Leveridge (1987) show mainly acidic with a mixture of felsic and basic sources in Nfaitok and Ngeme formations. The acidic signature is mainly from Nfaitok Formation, while the mixed source detected in Ngeme and Nfaitok formations points to the various sources of these formations.

**Figure 11:**
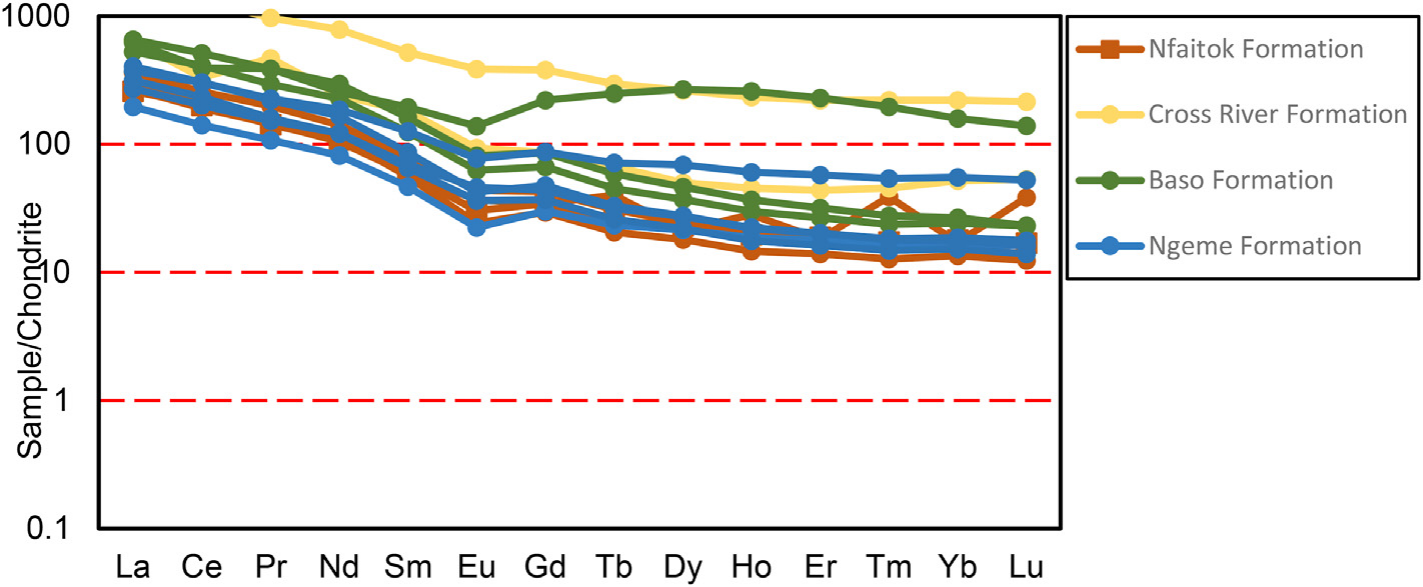
Chondrite normalized REE patterns of the different formations of the Mamfe Basin (McDonough and Sun, 1995).

Moreover, the Co/Th vs La/Sc ratios diagram locates samples from Ngeme and Nfaitok formations in the felsic igneous rock area, confirming this provenance (Figure 10b). Furthermore, previous studies from Taylor and McLennan (1985), Wronkiewicz and Condie (1987), Madhavaraju et al. (2002) reported that REEs, HFSE, Zr, Hf, Yb and Nb, some transition trace elements (TTE) such as Sc, Co, Cr and Ni along with Al, Ti and Th remains stationary during diagenesis, such constituents are essential in the understanding of source rock composition. The La_N_/Yb_N_ ratio represents the fractionation indice, an expression of the enrichment of the LREEs over HREEs. In the present study, the La_N_/Yb_N_ ratio average values vary from 10 to 17 and indicate that HREE are strongly depleted in favor of LREE. This ratio with the Eu anomaly appears as estimated indicator to depict REEs profiles and may be applied to infer provenance. The Eu/Eu∗ ratio (where Eu∗ is europium anomaly) is commonly useful to assess the source rock composition (Kutterolf et al., 2008; Dabard and Loi, 2012). In our study, the REEs normalized against chondrites pattern confirms the significant enhancement of LREE and decrease of HREE (Figure 11) with pronounced negative Eu anomaly indicative of felsic provenance.

**Figure 12:**
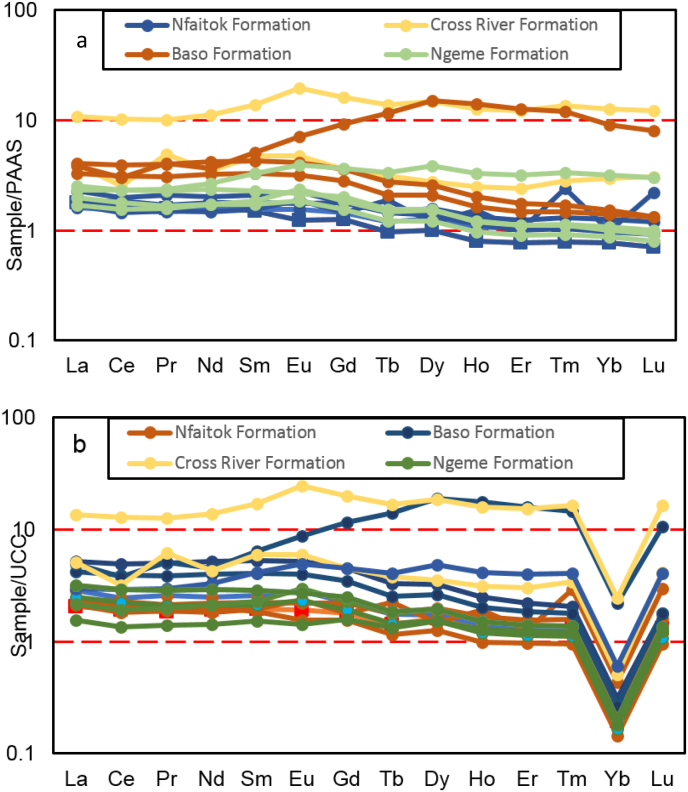
(a) Post-Archean Australian Shale (PAAS) and Upper (b) Continental Crust (UCC) normalized REE patterns of the different formations studied after Taylor and McLennan (1985).

The diagram of UCC (Upper Continental Crust) normalized REEs profiles of the Mamfe basin is illustrated in Figure 12b after Taylor and McLennan (1985). The diagram reveals a marked Yb negative anomaly suggesting igneous fragmentation under highly reducing conditions (Hsu and Crozaz, 1995).

The cerium anomaly Ce is of great significance because it provides important information to access sediment provenance. The Ce anomaly is expressed by Ce/Ce∗, Ce∗ is obtain by chondrite normalized REEs pattern. The calculation of Ce/Ce∗ anomaly is provided in Table 4. The Ce/Ce∗ anomaly concentration varies from 0.72 to 0.85 in Ngeme, 0.59 to 1.45 in Nfaitok, 0.72 to 0.77 in Baso and 0.74 to 0.78 in Cross River formations. Murray et al. (1990) reported Ce/Ce∗ anomaly value between 0.79 and 14.5, referring to deposition on a continental margin. Furthermore, the chondrite normalized REEs profile shows a slightly positive Ce anomaly.

On the other hand, the Gd_N_/Yb_N_ ratios reflect the type of bedrock as well as the content of continental crust (Taylor and McLennan, 1985) Gd_N_/Yb_N_ ratio is high in Archean crust with a value above 2.0 in sedimentary rocks, while its value commonly varies around 1.0 to 2.0 in post-Archean materials (McLennan and Hemming, 1992). Only samples from Cross River Formation have a value lower than 2.0. In contrast, the other formations have an average value of Gd_N_/Yb_N_ above 2.0, indicative that the post-Archean rocks were the originating material of the sediment of Ngeme, Nfaitok and Baso formations (Table 4).

Table 5 shows that in Ngeme Formation Ni, a significant correlation with Sc, Rb, Zr, Nb, Sr, Cs, Ba, Hf, Ta, Pb, Th, U, ƩREE (r = 1). Whereas in Nfaitok Formation, Sc has a significant positive correlation with Hf, Ni, Zn, Rb, Zr, Th, ƩREE (r = 0.87). Additionally, samples from Ngeme Formation, the concentration of Ni and V have any notable correlation with some detrital constituents such as Nb, Hf, Zr and Th (Tribovillard et al., 1994; Böning et al., 2004) (Table 5). Th is considered as good proof for detrital supply in the depositional area due to its conservative geochemical behavior (Cochran et al., 1986; Pollack et al., 2009). Th exhibits a good correspondence with Sr, Nb, Cs, Ba, Hf, Ta, Pb.

### 5.5 Weathering intensity and chemical maturity

Numerous studies reported that chemical weathering influence the chemical composition of the siliciclastic sediment (Nesbitt and Young, 1982, 1984; Johnson et al., 1988; McLennan et al., 1993; Fedo et al., 1995). During this process, Ba and Al are stable in the sediments compared to Ca, Na and Sr that are taken away (Fedo et al., 1996; Nath and Kunzendorf, 2000). According to Nesbitt and Young (1982), The CIA is a good proxy for evaluating sediments alteration. It is expressed by CIA = 100 X (Al_2_O_3_/(Al_2_O_3_+CaO∗+Na_2_O + K_2_O)). The various component of the later formula is in molar proportions. Which CaO∗refers the quantity of CaO added into silicate fraction of sample. The calculation in this study based on McLennan et al. (1993) approach to discern as well to measure the CaO component in silicate and a non-silicate fraction. The expression of CaO∗.formula is CaO∗ = CaO-10/3(P_2_O_5_). In the processing, when the rest amount of mole is inferior to Na_2_O content, the value is retained as content of CaO in silicate. Otherwise, when the rest amount of mole is higher than Na_2_O value, then Na_2_O is used as CaO content in silicate. This is due to the fact that Ca is generally affect primly compared to Na in the course of alteration.

According to McLennan et al. (1993) and Fedo et al. (1995), the CIA values ranging between 45-55 imply weak weathering. In contrast, values of 100 denote deep alteration with full elimination of alkali and alkaline earth constituents with significant rate of Al_2_O_3_ contents. The CIA values of this study in Table 1 is comprised from 47.96 to 61.99 (mean 55.41) for Ngeme, 2.10 to 70.26 (mean 40.26) for Nfaitok, 64.29 to 66.81 (mean 65.75) for Baso and 62.75 to 63.61 (mean 63.18) for Cross River formations indicating a weak to medium level of weathering of material. The CIA values scale is integrated into a trivariate diagram of Al_2_O_3_–CaO–Na_2_O–K_2_O or A–CN–K (Figure 14). This A–CN–K chart shows two differentiated trends. The first trend close to A-CN axis suggests that most of the sample sustained a weak rate of alteration. The second short trend parallel to the A-K axis indicates a medium rate of alteration of few samples in the field of illite and muscovite. This result may result from rapid burial with a shorter exposure time and shorter transport distance before deposition. Low to moderate weathering depict a hot and moist climate in the basin.

Cox et al. (1995) reported that sediment with an index of compositional variability ICV >1 is non-mature and refers to tectonically active settings. While those with ICV <1 are mature and deposited in a tectonically quiescent or cratonic setting, where sediment recycling is active. Accordingly, in this study, the entire ICV >1 in Ngeme, Nfaitok, Baso and Cross River formations implying that samples from MSB are immature and deposited in a tectonically active setting.

### 5.6 Paleoenvironment and paleoclimate

According to Wronkiewicz and Condie (1987), climate and tectonic activity control the alteration of mineral process. The high level of mineral alteration matches the low grade of tectonic and climate fluctuation to hot and moist conditions conducive to mineral degradation in the deposition area (Jacobson and Blum, 2003). The SiO_2_/(K_2_O + Na_2_O + Al_2_O_3_) ratio is widely used to discriminate paleoclimate conditions after Suttner and Dutta (1986), because during the alteration phase, K_2_O, Na_2_O and Al_2_O_3_ are easily attacked and leached compared to SiO_2_. The plotted diagram of SiO_2_/(K_2_O + Na_2_O + Al_2_O_3_) ratio from this study places the whole samples in the arid and sub-arid field (Figure 15), reflecting the climatic conditions during the deposition.

Furthermore, the Ga/Rb vs Sr/Cu ratios for fine-grained sediments is widely referred to constrain the paleoclimate regime (Xie et al., 2018). According to Beckmann et al. (2005), Roy and Roser, 2013a, Galium is most often associated with fine-grained aluminosilicate portion and is high in kaolinite, mirroring hot and moist climate conditions. In contrast, Rubidium is linked with illite and indicates poor intensity of alteration, referring to warm and cool weather conditions (Roy and Roser, 2013a). Therefore, the Ga/Rb value decreases in sediments (Roy and Roser, 2013b) in cold and dry climate conditions, whereas the Ga/Rb value increase in warm and humid climate conditions.

Xu et al. (2017) documented that high Ga/Rb and low Sr/Cu proportion value in sediments usually suggest dry and moist weather conditions. The Sr/Cu proportion value between 1.3 to 5.0 in deposits is thought to reveal humid conditions, while values above 5.0 indicate dry conditions (Sarki Yandoka et al., 2015; Xu et al., 2017). The plot diagram of Ga/Rb and Sr/Cu proportion for this study (Figure 16) shows low Ga/Rb and Sr/Cu ratios values respectively range from 0.14 to 0.3 for Ga/Rb and 0.1 to 4.5 for most samples from Ngeme Formation. Whereas in Nfaitok Formation, most samples have a high Sr/Cu ratio (5–15) and display on either side of the threshold. This result reveals that most Ngeme Formation sediments have undergone warm and humid climate conditions. In contrast, most deposits from Nfaitok experienced arid to semi-arid climate conditions with an intermittent humid period.

Moreover, the distribution of some ratios in the sediment, including Sr/Ba, Sr/Cu, V/Ni ratios and ƩREE in selected sections from Ngeme and Nfaitok formations, is shown in Figure 17. The Sr/Ba ratio is moderate with relatively low values and increase slightly in Ngeme and Nfaitok formations indicative of oxic conditions. The Sr/Ba ratio trends in Ngeme and Nfaitok formations are stable and increase somewhat on the top of profiles. The V/Ni ratio trends rise in Ngeme and Nfaitok formations. Increase of Sr/Ba, Sr/Cu, V/Ni ratio in Ngeme and Nfaitok sediments pointing to increase of reducing condition. The ƩREE concentration is important and exhibits a stable trend that grows in a few points along with the profile in Ngeme and Nfaitok formations, implying that the input of terrigenous sedimentary rock was significant and consistent during the depositional time.

Redox conditions are essential to recognize the sediment deposition in marine and continental environments. According to McKay et al. (2007), the high concentration of the following elements ratios including U/Th, Ni/Co, Cu/Zn, (Cu + Mo)/Zn and V/Cr, applied for assessing paleoredox states. Jones and Manning (1994) reported that the U/Th ratio is significant in mudstones in contrast to sandstones. Numerous studies including those of Hallberg and Johnston (1976) and Jones and Manning (1994) show that U/Th ratios under 1.25 implies oxic state of sediment, whereas values of U/Th ratio over 1.25 denote suboxic and anoxic conditions. The U/Th ratios in Mamfe samples rank for 0.07 to 1 (mean of 0.89), indicative of oxic conditions.

Furthermore, the Ni/Co ratio is helpful in determining the source area's redox states (Bjorlykke, 1974; Dypvik, 1984; Dill, 1986; Brumsack, 2006; Nagarajan et al., 2007). The Ni/Co ratio less than 5 indicates oxic conditions, the Ni/Co ratios more than 5 implies suboxic and anoxic states in the deposition area (Jones and Manning, 1994). The Ni/Co ratio of the studied samples ranges from 0.7 to 7.6 (average of 1.3), implying that the sediment was mainly deposited in an oxic environment, including minor intervals with prevailing suboxic or anoxic conditions.

The V/Cr ratio is utilized as indication of paleoxygenation (Bjorlykke, 1974; Shaw et al., 1990; Nagarajan et al., 2007; Madukwe, 2016). The dissolution of vanadium in surface waters, as well as its migration from seawater into sediments are mainly influenced by redox states. The V/Cr ratios value over 2 indicates anoxic conditions, whereas V/Cr values under 2 indicate more oxidizing conditions (Jones and Manning, 1994). The V/Cr ratios of the Mamfe sandstone range between 0.9 and 6.36 (mean 1.69), which indicate that sediment predominantly developed in an oxic area with some episodic phase of suboxic or anoxic conditions. Fingerprints of episodic anoxia are justified by sedimentary pyrite in organic-rich shale and depleted Yb in the sediment.

Based on Hetzel et al. (2009) investigation the value of the V/Sc ratio under 9.1 indicates oxic influence. The V/Sc ratios value of the Mamfe sandstone ranges between 3.67 and 19.1 (mean 8.41), suggesting oxic depositional conditions with influence of episodic anoxic conditions.

## 6 Diagenetic history

The MSB sediment consists mainly of detrital and neoformed quartz with an essential proportion of plagioclase and K-feldspar, with less than 20% igneous and metamorphic rock fragments. Plagioclases and microclines dominate feldspars; orthoclase feldspar is rare. These feldspars are partially altered to produce authigenic minerals, namely kaolinite, montmorillonite, sericite, calcite, pyrite, apatite, siderite, hematite and vivianite. They most often display cracks and fissures that are evidence of compaction. These cracks are filled with opaque minerals or alteration products. The rock fragments are polycrystalline quartz assemblages with crenulated contours. The main mafic mineral is micas which exhibit shapes in rods, elongated needles or flakes scattered in the rock. The opaque minerals represented by hematite, siderite, titanium dioxide, zircon, rutile and pyrite are widespread throughout the rock; they appear isolated in calcite cement or lodged within the other minerals. Phosphatic mineral consists of authigenic apatite and vivianite. Siliceous, clayey, calcareous and/or clay-siliceous cements occupy more and less important proportions depending on the formation. In the Cross River Formation, it occupies more than half of the rock. The matrix of the MSB rock from Ngeme, Nfaitok, Baso and Cross River formations consist of argillaceous and ferruginous products. Argillaceous matrix and calcite cement are derived from the weathering of plagioclase feldspar. Plagioclase dissolution is also recognized as a weathering product (Bloch & Franks, 1993), apparently resulting from early diagenesis from meteoric water (Morad, 1998).

The high proportion of feldspar in sandstone in the basin suggests that sediment is mineralogically sub-mature to immature. The grains occur more angular to subangular than subrounded in Ngeme, Nfaitok and Cross River formations indicative of the vicinity of the parental rock.

Diagenetic processes of these sediments include compaction, cementation, dissolution and replacement of some clay and neoformed minerals. Compaction in the basin is marked by deformed biotite within sub-parallel oriented mica flakes. In general, reduced porosity in the basin is due to the combined action of compaction and cementation. Two generations of calcitic cementation occur in sandstones of the Nfaitok Formation, namely early calcite cement and late calcitic cement enriched in magnesium, which developed secondary porosity in the sandstone. Some sandstone beds underwent early cementation by sparse pyrite and siderite in Nfaitok Formation. The basin sediments show two forms of calcites, calcic (CaCO_3_) and ferroan calcite (Fe, Ca (CO_3_))_2_. Siderite formed during late diagenesis of calcite, especially in Fe- and carbonate-rich fluids deficient in Ca ^2+^ cations. Mg and Mn could be intergraded into siderite composition during the late diagenetic stages.

During moderate to deep burial, calcite cementation was common in the basin, up to and at maximum burial depths. Detrital plagioclase and feldspar are the essential source of silicate diagenesis in sandstones. Partial dissolution of feldspar in sandstones occurred during early diagenesis.

Diagenetic substitutes are genuine minerals derived from detrital minerals and rock fragments. They consist of clay minerals such as kaolinite and montmorillonite, sericite and calcite derived from the partial alteration of feldspars. Chlorite originates from the recrystallization of detrital clays, and siderite derived from calcite during the late diagenesis stage, whereas fromboidal pyrite derives indirectly via iron monosulphide. In contrast, euhedral pyrite formed directly as precipitation from solution (Swalowicz, 1993), and neoformed quartz results from primary quartz outgrowths. Pettijohn et al. (1972) reported that the precipitation of calcite and dolomite in sandstone is linked to Ca and Mg significance as well as the pH of the depositional area.

In contrast, Fe and Mn rich carbonates are also dependent on the environment's oxidation-reduction balance because Fe and Mn occur in carbonate only in its reduced form. The occurrence of authigenic iron minerals such as pyrite, hematite, siderite, and vivianite suggest reducing deposition conditions. Organic matter is thought to be the most reducing agent in charge of Fe state (Pettijohn et al., 1972). For these authors, siderite with reduced carbonates is significant in organic matter rich environment. Such settings are stable basins, or tidal or estuarine environments where biological activity is important. Further, Van Houten (1968) and Walker (1967) reported that most diagenetic hematite in sandstones originated from degradation of iron-minerals in warm and dry zones. All these diagenetic phenomena exposed above can lead to a decrease in permeability and an increase in the porosity of the reservoir rocks, which is a crucial factor for the accumulation of hydrocarbons.

## 7 Discussion

The considerable size of the basal conglomerates, more than 60 cm, and pebbles interbedded with sandstone strata in Ngeme Formation provides evidence of high energy water flow that have promoted extensive alteration and the potassium metasotism process enhanced by the formation of clay mineral as kaolinite and illite and replacement of plagioclase by potassium feldspar under hot and humid climate conditions. The interbeds sandstone probably resulted from this extensive weathering and was deposited during calm intervals. The sediments of Ngeme Formation are proximal alluvial fans deposited as overlapping lobes. Its provenance is from the nearby basement rocks (gneisses, granites), whose clasts have undergone a relatively short transport.

Samples from Nfaitok Formation consist of laminated carbonaceous shales, marlstone, volcaniclastic sediments, and limestone deposited in calm and hypersaline lacustrine or lagoonal environments. Haldar (2020) reported that lacustrine limestone is often enriched by oncoid arising from cyanobacteria wrapping of bioclastics or shells of gastropods and rock fragments. This description corresponds well to the limestone found at Nfaitok Formation and oncoids developed with ostracods in micrite. The abundance of micrite, organic-rich shales and planar lamination suggest low depositional energy characteristic of the pelagic environment. This environment concords with arid to semi-arid climate conditions with an intermittent humid period described in Nfaitok Formation (Bilobe et al., 2021). These sediments were likely deposited by wind. The laminated structure in Nfaitok Formation is probably the result of the movement of waves under the action of the wind. The humid period allows alteration of plagioclase to kaolinite.

The sediment in Baso Formation includes mudstone and coarse to pebbly sandstone suggesting the prodeltaic environment. This sediment underwent moderate alteration suitable to clay mineral product as montmorillonite and illite and significant k-metasomatism under semi-arid climatic conditions.

The Cross River Formation is characterized by greenish tabular sandstone, which alternates with mixed clay-rich sandy mudstone. The dominance of sediment supply suggests flood events with relatively high fluvial transport energy. The mixed sediment load suggests deposition in low sinuosity channels or broad alluvial plain. The cross-stratification signature is indicative of a period of strong agitation of the environment during the deposition. The importance occurrence matrix with micas, smectite, and basement clasts suggests intense weathering of volcanic and metamorphic source rocks. The prevailing climate was probably hot and humid with increased rainfall in the source area.

The new Cenozoic horizon made by fine-grained sandstone was deposited late during Neogen's calm period and underwent intense alteration, leading to smectite formation under humid climatic conditions (Bilobe et al., 2021). These sediments derive from felsic origin. This source rock is undoubtedly the granito-gneissic basement that surrounding the basin. Following all the above, the hypothesis of deposits of fluvial to lacustrine origin can be retained.

Sandstones from Baso and Cross River formations with their reddish-yellowish and purplish coloration indicate iron oxides and hydroxides (hematite, goethite), suggesting deposition in an oxidizing environment. The scarce storage of organic matter in the sediment of Cross River Formation is explained by its removal by this oxidation conditions that prevailed as revealed from hematite concretions and rubrication of sediments. Whereas the dark clayey siltites from Nfaitok Formation, consisting of thin-grained laminated sediments, indicate a confined depositional environment, reflecting the anoxic conditions that prevailed in the bottom waters of the basin during the Barremian-Cenomanian time. Moreover, carbonates in the basin may have formed under this confined anaerobic environment conducive to the accumulation of carbonate ions. This anoxia condition in the basin was episodic and promoted sedimentary pyrite in organic-rich shale and depleted Yb in the sediment.

**Figure 13:**
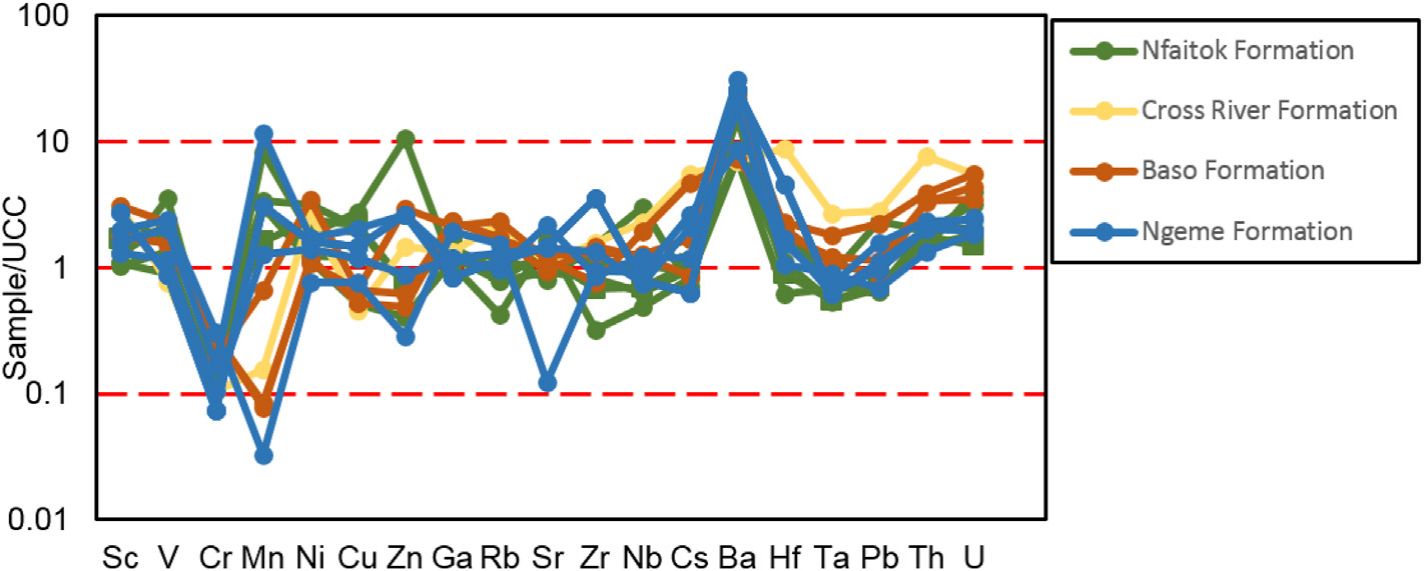
Distribution of some selected trace elements against Upper Continental Crust (UCC) of the different formations studied. UCC values are from Taylor and McLennan (1985).

The plot of major, trace and REEs in this study, including the discrimination function F1 and F2 as well as La/Th vs Hf and Co/La/Sc ratios (Figures 9 and 10), imply that these sediments are dominantly from igneous felsic (acid plutonic and volcanic rock) and intermediate igneous source (andesite) with a minor contribution of mafic and polycyclic continental sedimentary rocks. Sediments from Ngeme, Nfaitok, Baso and Cross River formations reflect a felsic provenance source, while those from Ngeme and Nfaitok formations reflect an intermediate source. The few mafic source occurrence includes sediment from Nfaitok Formation, and polycyclic quartzose provenance includes sediment from Ngeme Formation. This felsic provenance agrees with the chondrite normalized REE profile (Figure 11) which shows enhancement of LREE with Eu negative anomaly confirming the dominant felsic composition of the source rock. Furthermore, the high concentration of LREE (Figure 11) Th and Rb (Figure 13) confirm that these sediments carry a signature from continental arc magma.

**Figure 14:**
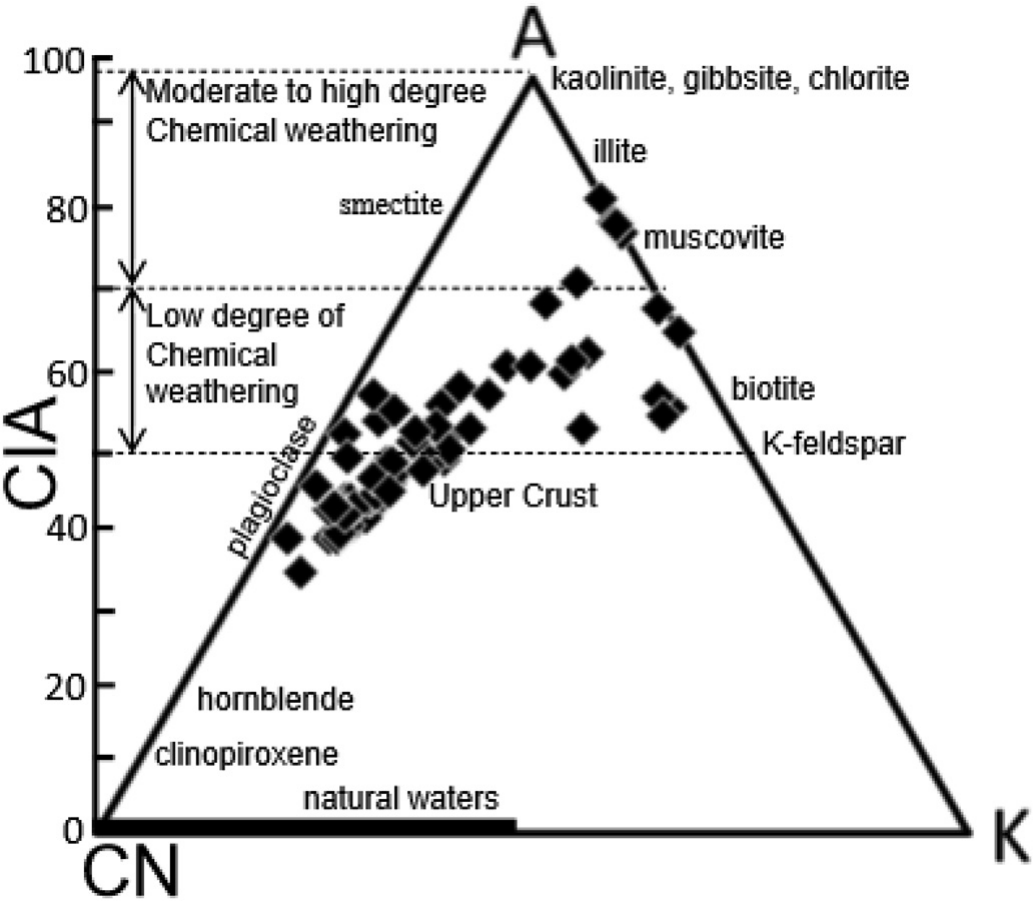
Ternary plots of molecular proportions of Al_2_O_3_-(Na_2_O + CaO∗)-K_2_O or A–CN–K diagram of the Mamfe samples with the CIA. Scale shown on the left according to Nesbitt and Young (1984).

**Figure 15:**
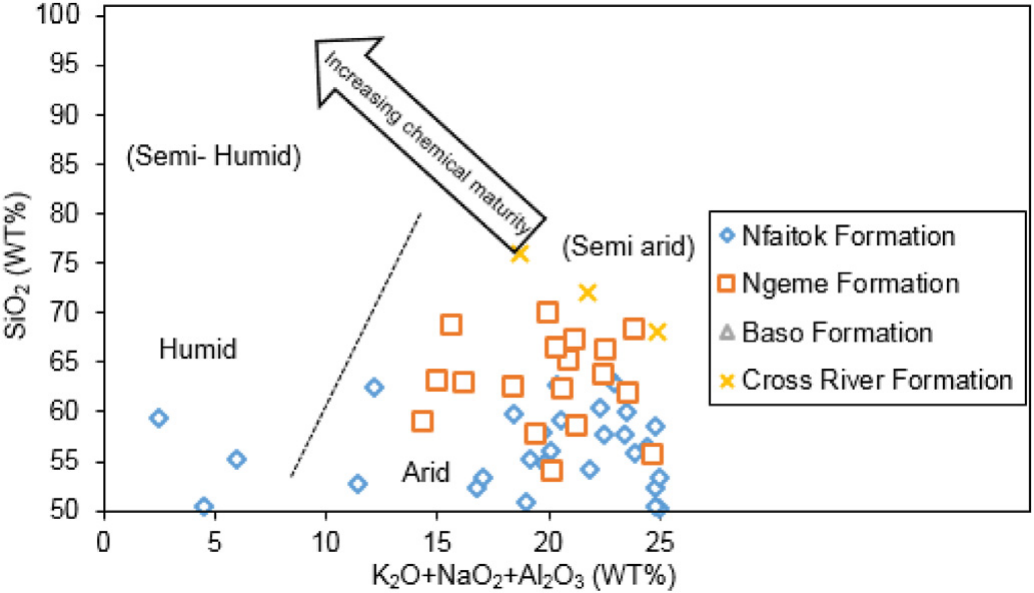
Bivariate plot of SiO_2_ versus K_2_O + Na_2_O + Al_2_O_3_ to discriminate paleoclimatic conditions during the deposition of the Mamfe sediment after Suttner and Dutta (1986).

**Figure 16:**
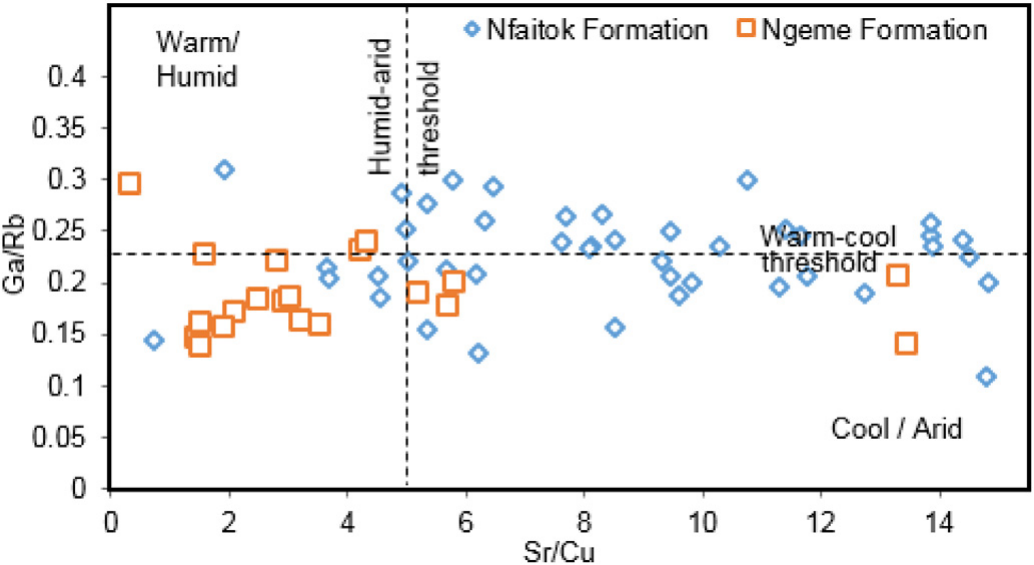
Cross plot diagram of Sr/Cu vs. Ga/Rb showing paleoclimatic variations after Xie et al. (2018).

**Figure 17:**
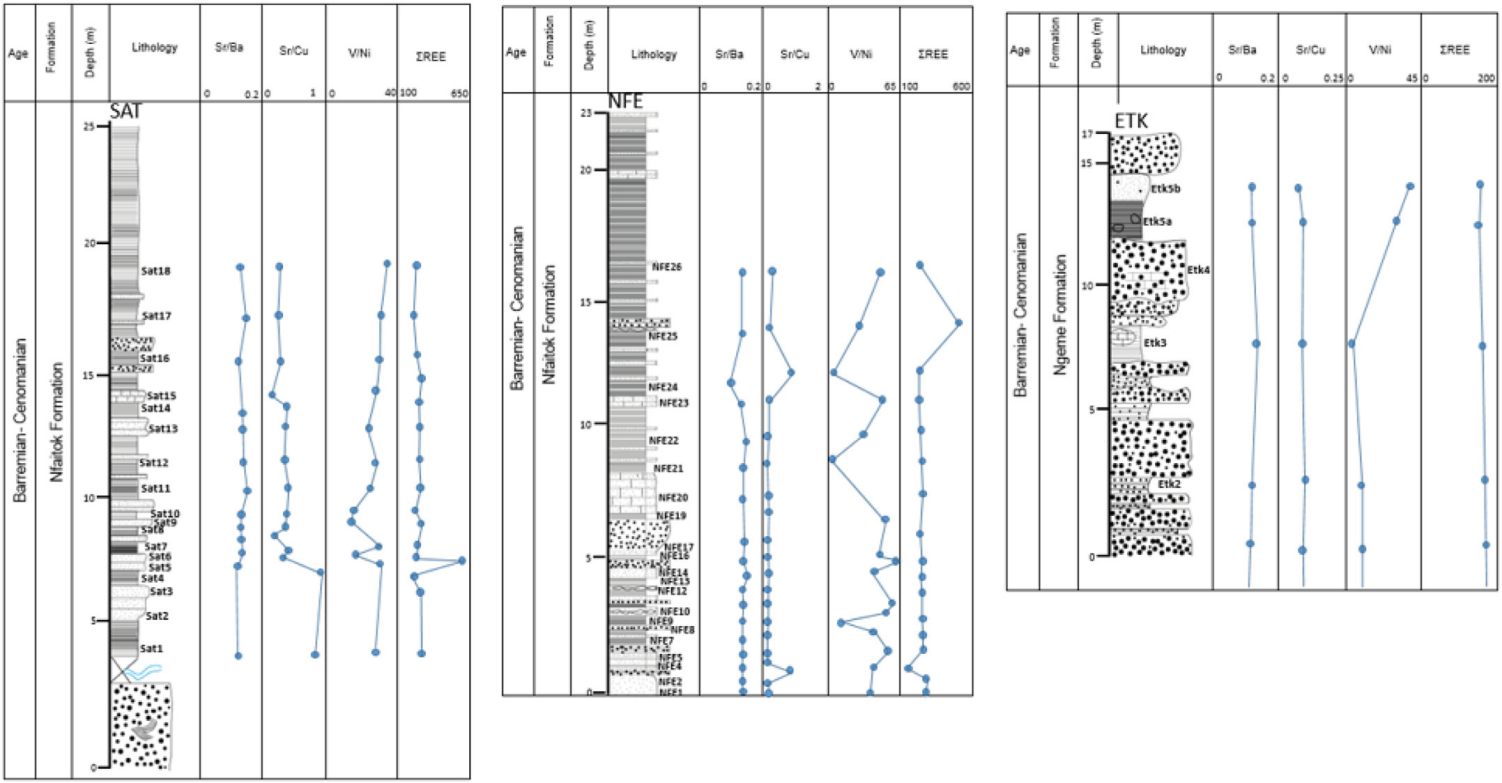
Stratigraphic distribution of trace elements and REEs in Nfaitok and Ngeme Formations.

On the other hand, the increase of P_2_O_5_ in the sediment of Nfaitok Formation (Figure 5 a and b) along with the precipitation of phosphate minerals such as fluorite and apatite seem to have taken place under basic conditions with the influence of seawater by the mixing of meteoric water and seawater, which enhanced the transition from acid to basic pH conditions. This composition probably explains the impact of low-rate mafic sediment recorded below in Nfaitok Formation. The presence of oncolithe in this formation confirms the lacustrine paleoenvironment of deposition.

This provenance reflects the tectonic regime in the basin, namely a passive margin and oceanic island Arc (Figure 8) since the continental crust is prone to intermediate or felsic composition (andesite, diorite). The active continental margin is favorable to forming the new igneous rocks due to tectonic activity (volcanoes). This scenario can explain the mixture of felsic source, which is predominant and linked to a continental plate. Thus, intermediate source rock is probably linked to the continental island arc, reflecting a mixture of mantle and continental crust material.

The geochemical composition of the investigated samples based on Fe_2_O_2_/K_2_O vs SiO_2_/Al_2_O_3_ ratios and the plot of log (Na2O/k2O) against. (SiO2/Al2O3) reveals that samples from Ngeme, Nfaitok and Baso formation are greywackes, shale and Fe-shale. In contrast, those from Cross River Formation consist of arkose. This difference in composition can explain that sediments from Cross River are less altered than those of the other formation. These arkoses were probably developed under arid to semi-arid conditions with a low rate of mineral alteration. The arkose in Cross River Formation is attributable to alluvial fan or fluvial apron of the parent rock in association with gypsum and other evaporitic minerals.

The CIA values from this study indicate weak to medium alteration (Nesbitt and Young, 1982). CIA values reflect cold and dry conditions (Fedo et al., 1995). This result concords with the bivariate plot of SiO_2_ versus K_2_O + Na_2_O + Al_2_O_3_ (Figure 8) which suggests that sediment was deposited under arid to semi-arid conditions. On the A–CN–K diagram (Figure 14), the primary tendency of silicate alteration of our samples displays preferential leaching of K_2_O (potassium feldspar, micas and clays mineral) and relative enrichment of CaO and Na_2_O (plagioclase feldspar and calcite). The linear alteration suggests a stable phase of mineral degradation (Nesbitt et al., 1997; Nesbitt and Young, 2004). This result may result from quick burial with a shorter exposure time and shorter transport distance before deposition.

**Plate 1:**
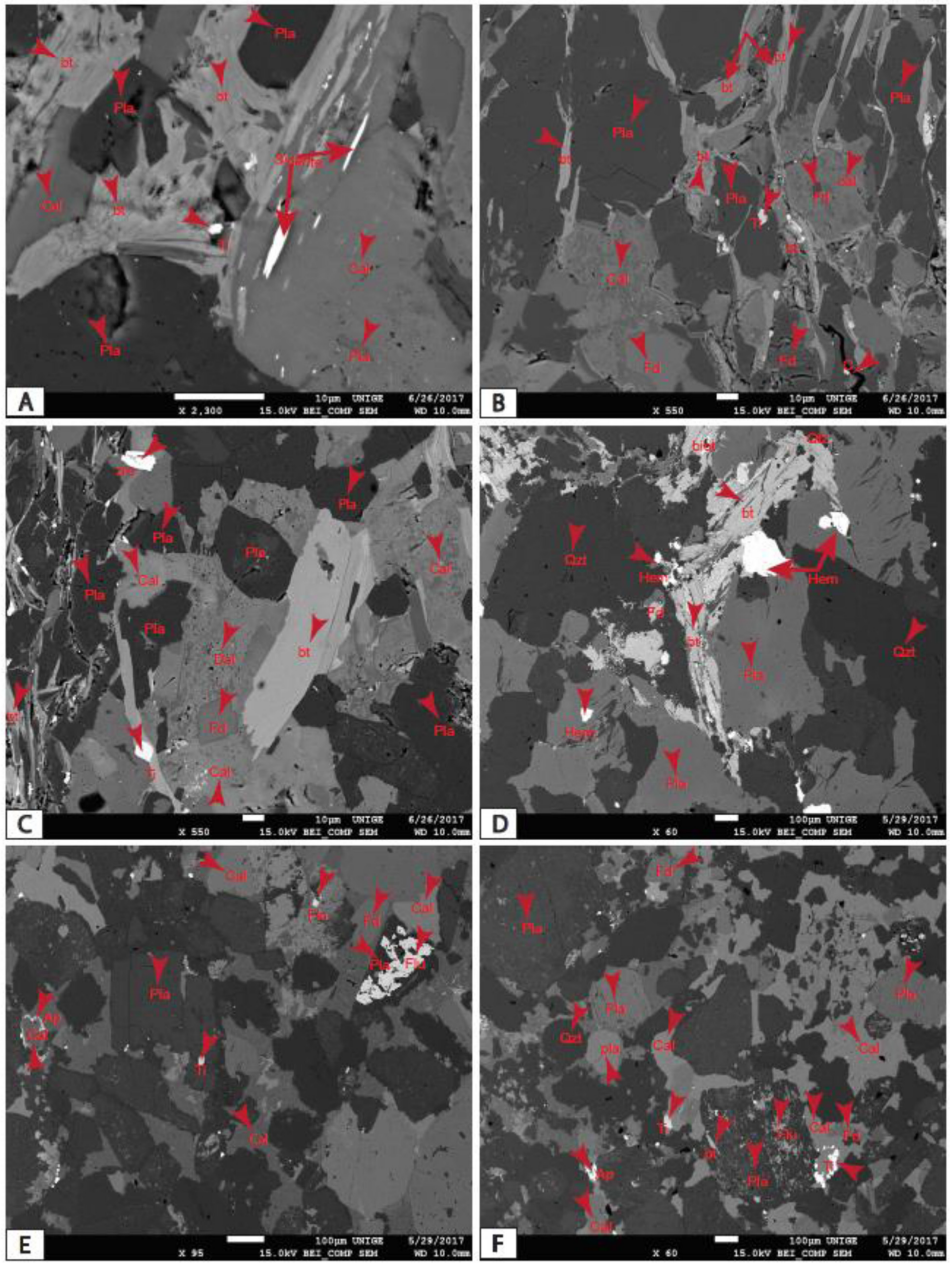
A) Fracture porosity of mica flake, plagioclase. Note the small inclusion of siderite and plagioclase in calcite mineral; B) Compaction of mica flake mineral and fractured plagioclase in calcite cement. C) Detrital quartz, plagioclase, feldspar and biotite in calcite cement, two generative of cement is present dolomitic cement present porosity. Biotite minerals present cross-linked contours indicative of rapid diagenetic process. D) Compaction of Detrital quartz feldspar, plagioclase, biotite and hematite. E) Detrital mineral in calcite cement, titanium, apatite, intrusion of fluorine in White cover plagioclase. F) Detrital mineral in calcite cement.

**Plate 2:**
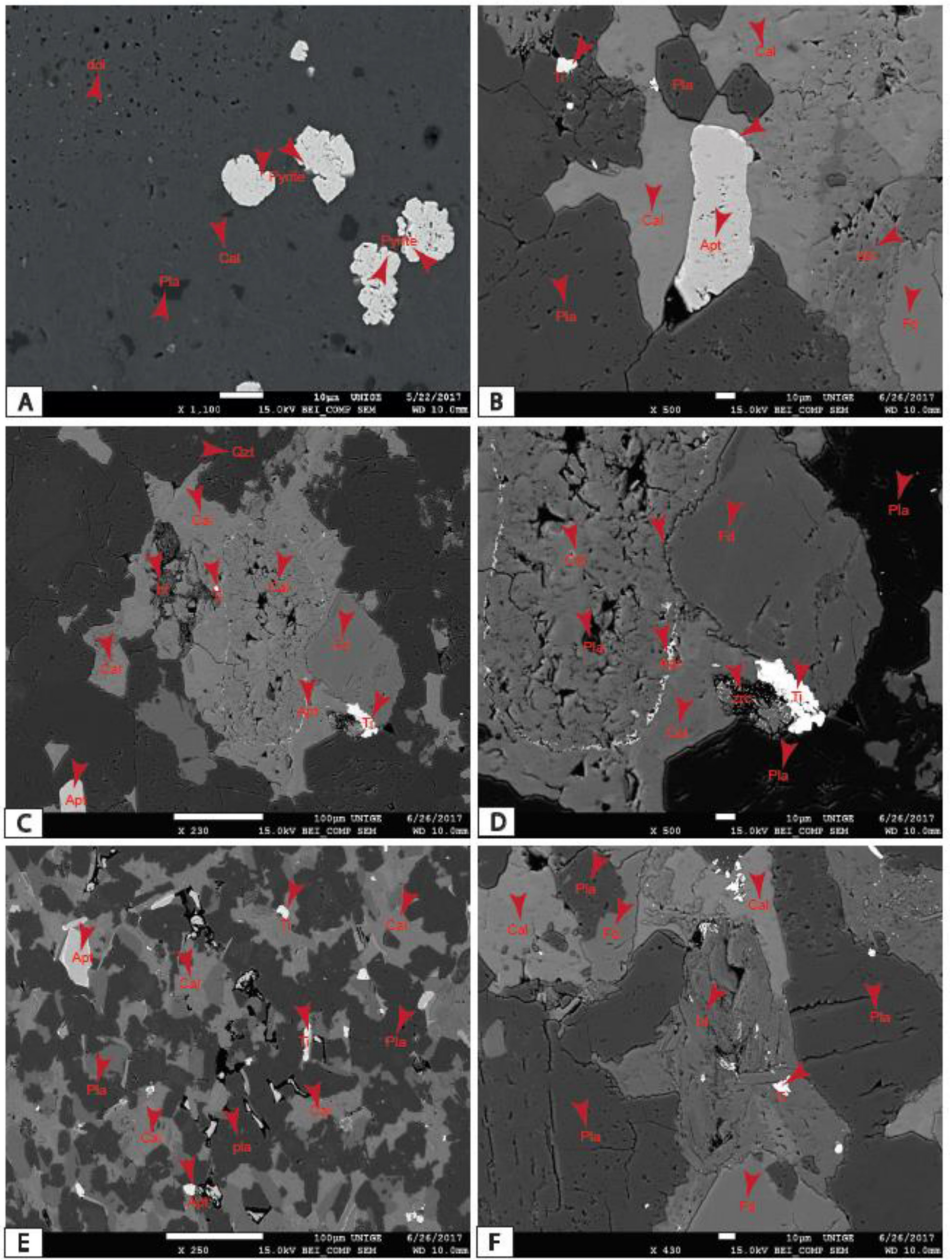
A) *Authigenic framboidal* pyrites and plagioclase inclusions within calcite mineral; B) Overgrowths of apatite grain, fractured plagioclase with titanium inclusion, feldspars with a reticulate edge in calcite cement. C, D) Feldspar mineral with reticulate shape suggesting post-depositional formation of the calcite cement; E) Apatite, quartz, plagioclase, titanite in calcite cement; F) Fracture porosity created within plagioclase and biotite in calcite cement.

**Plate 3:**
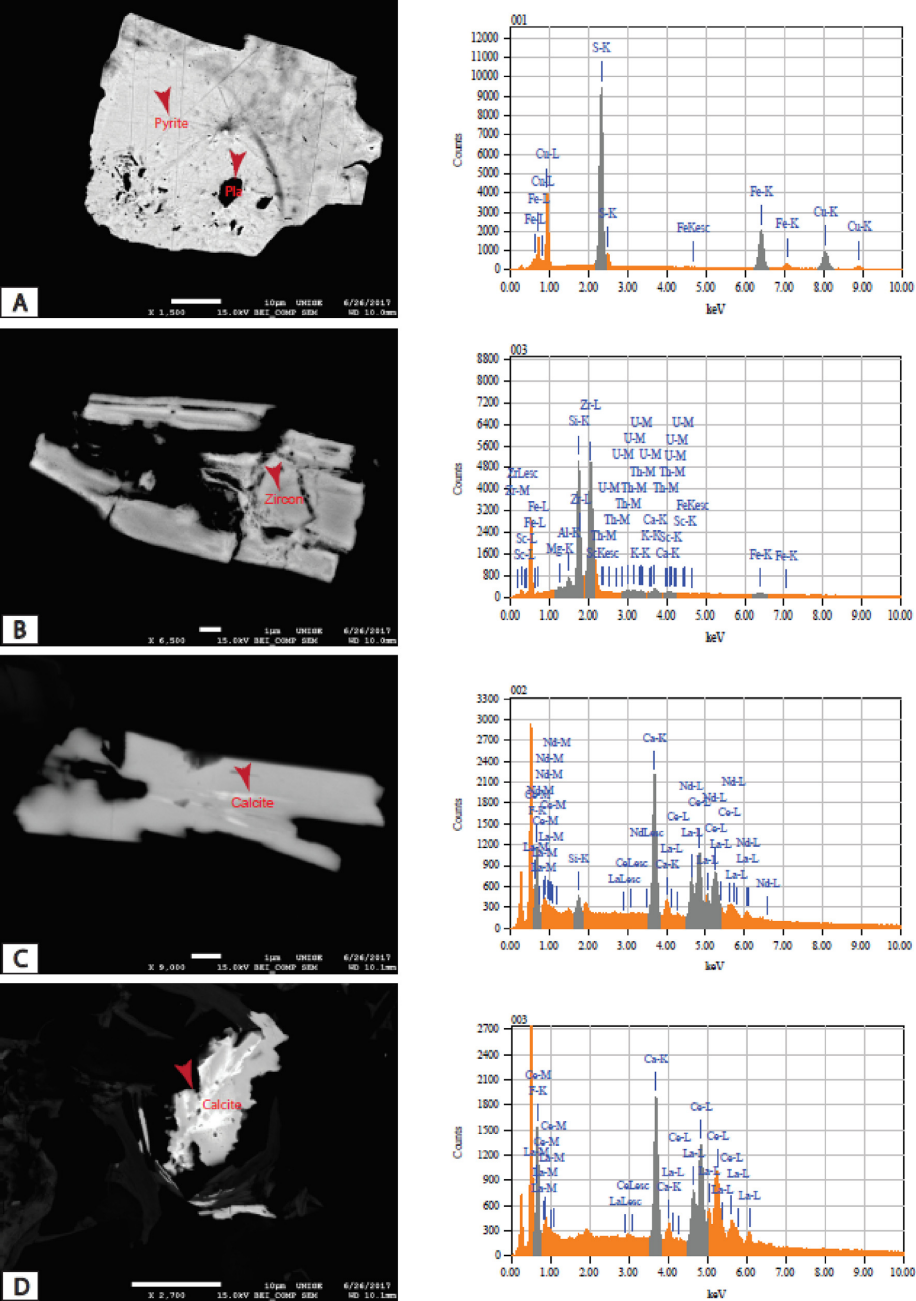
SEM view of images studied samples from the Mamfe Basin: A)- An altered pyrite mineral with small feldspar inclusion in dark; B)- Metamict zircon mineral enriched in thorium, uranium, iron, scandium, magnesium and aluminum. This enrichment suggests that metamorphic fluids transported uranium and thorium; C, D)- Altered authigenic calcite skeleton rich in neodymium, lanthane and cerium with feldspar inclusion suggesting alteration by aqueous-saturated phosphate solution (monazite) from phosphatic pegmatites or igneous, metamorphic or vein filling rock weathering.

**Plate 4:**
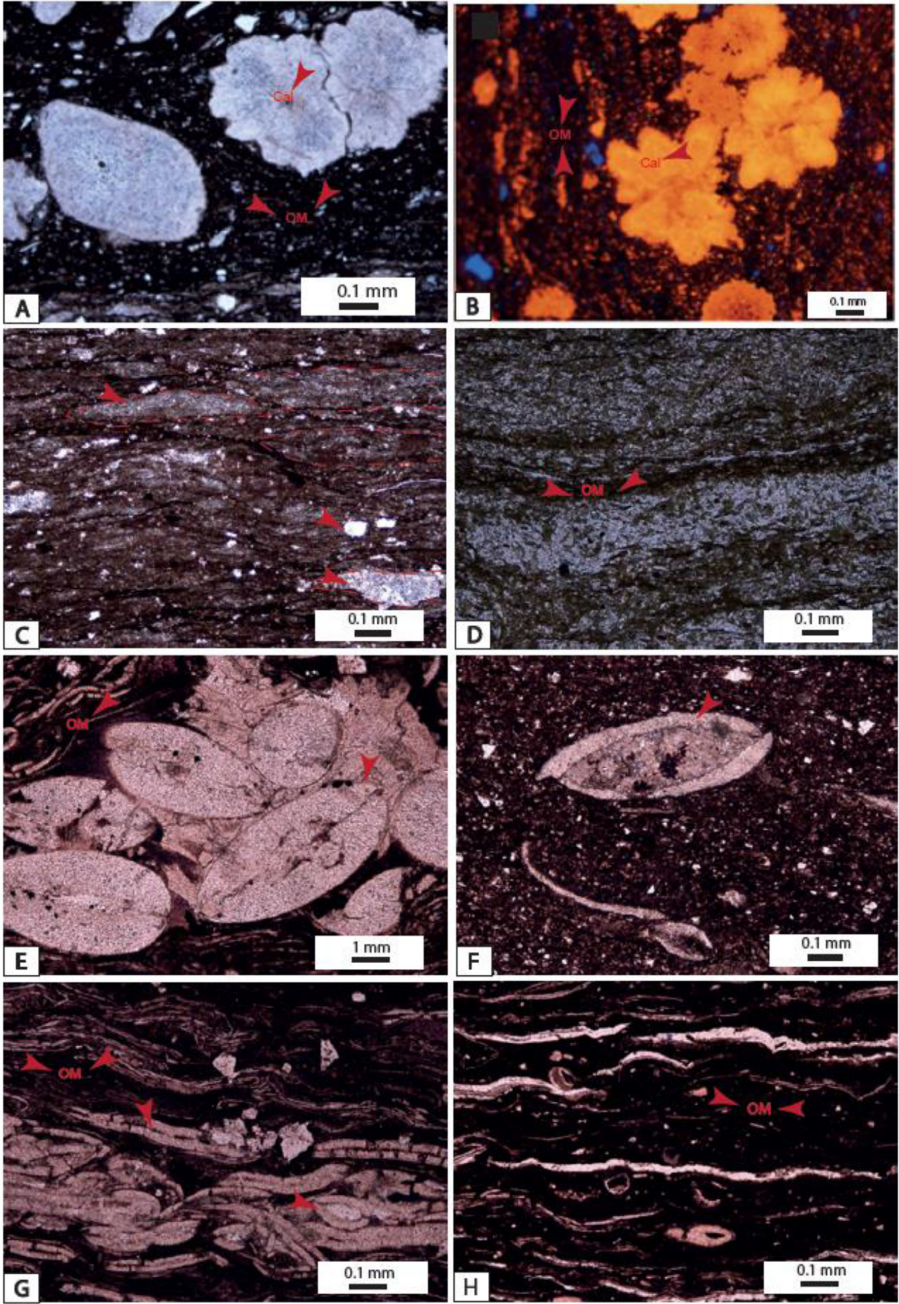
A, B) Mudstone with calcite concretion; C) Flaser bedding in calcitic mudstone; D) Laminated mudstone rich in OM white layer is quartz rich; E, F) Ostracods in mudstone; G, H) Open skeleton sponge framestone.

**Plate 5:**
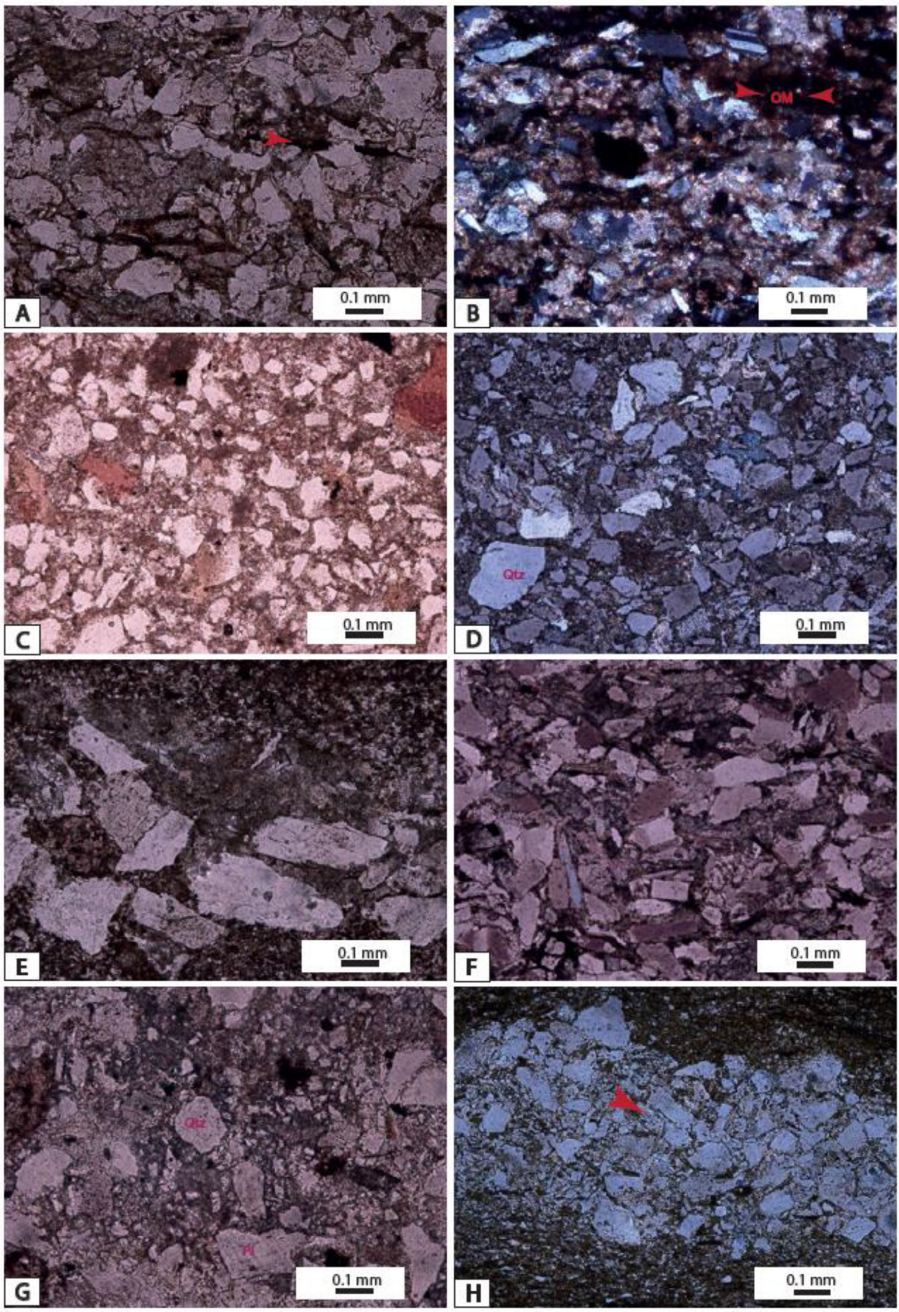
A, B) Ferruginous sandstone C) Sandstone in a ferruginous matrix; D) Greywacke in fine argillaceous matrix; E, F) Subangular sandstone; G) detrital grains in fine matrix H) Detrital clasts in mudstone matrix.

**Plate 6:**
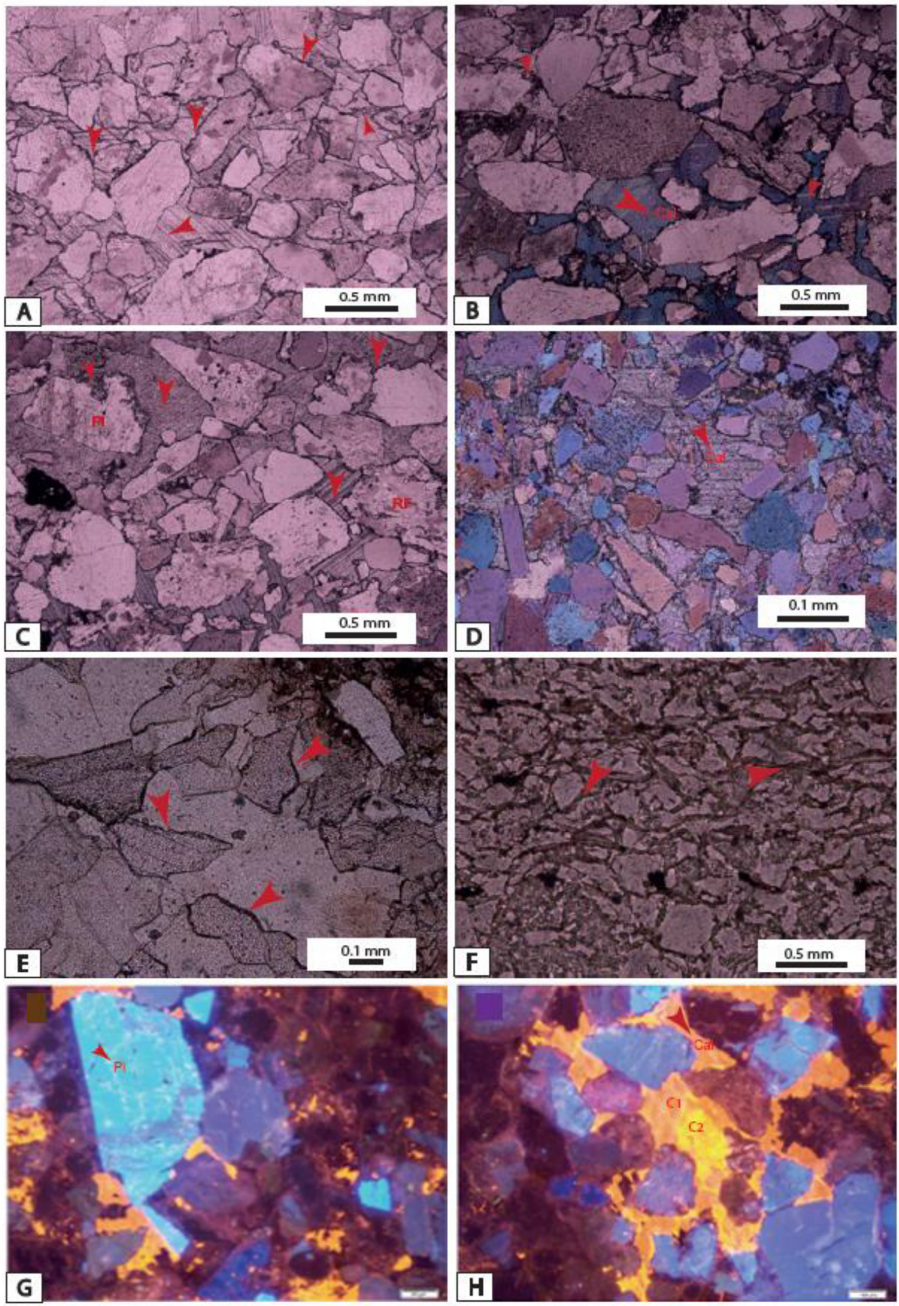
A, B, C, D) Calcareous corroded sandstone E, F) Subeuheudral calcite grains created elongated pore; G, H) Cathodoluminescence view of calcareous sandstone with 2 generation of cementation C1 and C2.

The ICV values range from this study are up to 1 (Table 1) and indicate that sediments from Ngeme, Nfaitok Baso and Cross River formations are compositionally immature in the basin. This later result is consistent with the high proportion of feldspars observed in deposits (Plates 1 and 2Plates 3, 4, 5,6).

The high rate of CaO and MgO (Figure 5b) is due to associated carbonate minerals or dolomitization. Whereas the enrichment of Fe_2_O_3_ reflects the influence of iron minerals (hematite, rutile), and enhancement in TiO_2_ is caused by occurrence of opaque minerals (Figure 5a). The enrichment of P_2_O_5_ (Figure 5b) may reflect the presence of secondary stages including apatite and monazite.

## 8 Conclusion

The MSB sediments derived from multiple sources, including felsic source, which is dominant, followed by intermediate source with a minor influence of mafic source. Sediment was subjected to weak and medium alteration during the diagenetic state.

The sediments of the Ngeme Formation are proximal alluvial fans deposited as overlapping lobes in a substantial dynamism period, which alternate with calm periods that have allowed the deposition of fine sediments. Its provenance is from the nearby basement rocks (gneisses, granites), whose clasts have undergone a relatively short transport. Sediments of the Nfaitok Formation were initiated in a calm and hypersaline lacustrine environment. Whereas the Baso Formation originated in a prodeltaic environment. In Cross River Formation, sediments were deposited under high energy conditions in a fluviatile environment. The new Cenozoic fine-grained horizon was deposited in a calm environment in a lacustrine environment.

The sedimentation was developed in arid to semi-arid conditions, corresponding to weak chemical maturity, suggesting that sediments were likely transported by wind and derived from multiple source area.

The high MnO and CaO content in sediment and Na_2_O in shale implies low leaching of plagioclase and immaturity of the basin's sediment. The deposits originated dominantly from passive continental margin and island arc.

The occurrence of sulfur minerals such as barite and authigenic pyrite suggests variation between oxic and anoxic conditions. Fingerprints of episodic anoxia are justified by sedimentary pyrite in organic-rich shale and depleted Yb in sediment.

## Declarations

### Author contribution statement

Jeanne Armelle Bilobe: Conceived and designed the experiments; Performed the experiments; Analyzed and interpreted the data; Contributed reagents, materials, analysis tools or data; Wrote the paper.

John Takem Eyong: Contributed reagents, materials, analysis tools or data; Wrote the paper.

Elias Samankassou: Conceived and designed the experiments; Contributed reagents, materials, analysis tools or data; Wrote the paper.

### Funding statement

This work was supported by the Swiss Government Excellence Scholarships for Foreign Scholars (2016.109), The University of Geneva (The Foundation Ernst & Lucy Schmidheiny) and the Société de Physique et d’Histoire Naturelle de Genève (Bourse Augustin Lombard).

### Data availability statement

Data included in article/supp. material/referenced in article.

### Declaration of interests statement

The authors declare no conflict of interest.

### Additional information

No additional information is available for this paper.
